# *CRB1*-Associated Retinal Dystrophies: Genetics, Clinical Characteristics, and Natural History

**DOI:** 10.1016/j.ajo.2022.09.002

**Published:** 2023-02

**Authors:** Malena Daich Varela, Michalis Georgiou, Yahya Alswaiti, Jamil Kabbani, Kaoru Fujinami, Yu Fujinami-Yokokawa, Shaheeni Khoda, Omar A. Mahroo, Anthony G. Robson, Andrew R. Webster, Alaa AlTalbishi, Michel Michaelides

**Affiliations:** 1 Moorfields Eye Hospital (M.D.V., M.G., K.F., S.K., O.A.M., A.G.R., A.R.W., M.M.), London, United Kingdom; 2 UCL Institute of Ophthalmology, University College London (M.D.V., M.G., K.F., Y.F.-Y., O.A.M., A.G.R., A.R.W., M.M.), London, United Kingdom; 3 Jones Eye Institute (M.G.), University of Arkansas for Medical Sciences, Little Rock, Arkansas, USA; 4 St John of Jerusalem Eye Hospital group, Jerusalem, Palestine (Y.A., A.A.); 5 Imperial College London (J.K.), London, United Kingdom; 6 Laboratory of Visual Physiology, Division of Vision Research, National Institute of Sensory Organs, National Hospital Organization Tokyo Medical Center (Y.F.-Y.), Tokyo, Japan; 7 Department of Health Policy and Management, School of Medicine, Keio University(Y.F.-Y.), Tokyo, Japan

**Keywords:** Retinal dystrophy, LCA, Early Onset Severe Retinal Dystrophy, RP, Macular dystrophy, CRB1, Gene therapy, Optical coherence tomography, Fundus autofluorescence, Genotype, Phenotype

## Abstract

**PURPOSE:**

To analyze the clinical characteristics, natural history, and genetics of *CRB1*-associated retinal dystrophies.

**DESIGN:**

Multicenter international retrospective cohort study.

**METHODS:**

Review of clinical notes, ophthalmic images, and genetic testing results of 104 patients (91 probands) with disease-causing *CRB1* variants. Macular optical coherence tomography (OCT) parameters, visual function, fundus characteristics, and associations between variables were the main outcome measures.

**RESULTS:**

The mean age of the cohort at the first visit was 19.8 ± 16.1 (median 15) years, with a mean follow-up of 9.6 ± 10 years. Based on history, imaging, and clinical examination, 26 individuals were diagnosed with retinitis pigmentosa (RP; 25%), 54 with early-onset severe retinal dystrophy / Leber congenital amaurosis (EOSRD/LCA; 52%), and 24 with macular dystrophy (MD; 23%). Severe visual impairment was most frequent after 40 years of age for patients with RP and after 20 years of age for EOSRD/LCA. Longitudinal analysis revealed a significant difference between baseline and follow-up best-corrected visual acuity in the 3 subcohorts. Macular thickness decreased in most patients with EOSRD/LCA and MD, whereas the majority of patients with RP had increased perifoveal thickness.

**CONCLUSIONS:**

A subset of individuals with *CRB1* variants present with mild, adult-onset RP. EOSRD/LCA phenotype was significantly associated with null variants, and 167_169 deletion was exclusively present in the MD cohort. The poor OCT lamination may have a degenerative component, as well as being congenital. Disease symmetry and reasonable window for intervention highlight *CRB1* retinal dystrophies as a promising target for trials of novel therapeutics.

Biallelic disease-causing variants in Crumbs homolog 1 (*CRB1*, MIM 604210) have been associated with a wide and complex range of phenotypes. The most commonly reported is Leber congenital amaurosis (LCA) or early-onset severe retinal dystrophy (EOSRD), where *CRB1* accounts for approximately 10% of all cases.^1^ This is followed in frequency by retinitis pigmentosa (RP),^2^^,^^3^ in which *CRB1* represents up to 6.5%.^4^ Other phenotypes include cone-rod (CRD),^5^^,^^6^ macular dystrophy (MD),^7^^,^^8^ foveal retinoschisis,^9^ and fenestrated slit maculopathy.^10^

One of the distinctive features of *CRB1* retinopathy can be the presence of nummular pigmented deposits,^11^ admixed with small yellow or white dots.^12^ Also, *CRB1* is commonly associated with preserved para-arteriolar retinal pigment epithelium (PPRPE),^2^^,^^13^ abnormally laminated and thickened retina,^14^ and peripheral exudative retinal telangiectasia (Coats-like vasculopathy)^15,16^—all of which can ultimately lead to retinal detachment and neovascular glaucoma.^5^ Nonretinal features have also been linked to this gene such as nanophthalmos,^17^^,^^18^ hyperopia,^13^^,^^19^ narrow anterior chamber,^20^ and optic disc drusen.^18^

*CRB1* was first identified in several unrelated individuals with RP and PPRPE.^21^^,^^22^ *CRB1* encodes a transmembrane protein with multiple epidermal growth factor–like and laminin A globular–like domains^15^ and is believed to have a role in retinal development as well as in long-term retinal integrity. Its primary function is in the maintenance of the zonula adherens junctions between photoreceptors, Müller glial cells, and the external limiting membrane.^23^, ^24^, ^25^ It also has essential roles in epithelial cell polarity and in the scaffolding complex, in vascular integrity, and is key to the preservation of an organized photoreceptor layer.^26^

*CRB1* is frequently reported as one of the most common causative genes for LCA/EOSRD.^27^, ^28^, ^29^ This has driven increasing efforts to develop animal models and treatments.^30^^,^^31^ As the preclinical work moves forward, it becomes imperative to understand the natural history of the disease. In this retrospective international study, we undertake deep phenotyping of the largest *CRB1* cohort as of this writing, report disease natural history, and explore potential endpoints for future interventional clinical trials.

## METHODS

The study protocol adhered to the tenets of the Declaration of Helsinki and was approved by the ethics committees of the participating institutions.

### PATIENT SELECTION AND GENETICS

The inclusion criterion for the current study was to have molecularly confirmed *CRB1*- associated retinopathy. This was defined as patients with an inherited retinal dystrophy (IRD) harboring 2 or more disease-causing *CRB1* variants. The patients were identified by reviewing the genetics database of Moorfields Eye Hospital (London, United Kingdom) and St John of Jerusalem Eye Hospital group (Jerusalem), and their records were subsequently studied.

Genetic testing was performed with various available methods, such as direct Sanger sequencing, next-generation sequencing–based retinal dystrophy gene panels, whole exome sequencing, and whole genome sequencing. In silico molecular genetic analysis was performed for all detected *CRB1* variants (transcript reference: NM_201253.3: ENST00000367400.3), and the detailed description is provided in the supplemental material (Supplementary Methods). Pathogenicity of each variant was classified mainly according to the guidelines of the American College of Medical Genetics and Genomics (ACMG).^32^^,^^33^ The cutoff value of allele frequency to apply PM2 (absence or very rare in the general population database) was 0.001. For the purpose of this study, an additional specification (likely pathogenic: 1 moderate [PM1-PM6] and 3 supporting [PP1-PP5]) to determine the verdict assessment results was applied to the ACMG classification.

### CLINICAL ASSESSMENT AND RETINAL IMAGING

All participants were seen by specialists in IRDs at referral sites. Clinical notes were reviewed, including family, medical, and ophthalmic history, best-corrected visual acuity (BCVA), refraction, slit lamp biomicroscopy findings, and funduscopy. BCVA was converted to logMAR for statistical analysis. Count fingers vision was given a value of 1.98 logMAR and hand motion, 2.28 logMAR; light perception and no light perception were 2.7 and 3 logMAR, respectively.^34^^,^^35^ Patients were categorized using the World Health Organization (WHO) visual impairment criteria, which defines no or mild visual impairment as BCVA ≤0.48 (6/18, 20/60), moderate impairment as BCVA >0.48 and ≤1.0 (6/60, 20/200), severe as BCVA >1.0 and ≤1.3 (3/60, 20/400), and blindness as BCVA >1.3.

Records of visual field were limited within our cohort; therefore, we only took into consideration central vision based on BCVA to classify patients. Low vision corresponds to patients with moderate and severe impairment. Asymmetric BCVA was defined as a difference ≥0.3 logMAR (equivalent to 15 ETDRS letters) between eyes. Refraction was undertaken by an optometrist for both adults and children, and spherical equivalent was calculated for refractive error.

When available, we also assessed additional testing such as color and autofluorescence retinal imaging, near-infrared reflectance, and optical coherence tomography (OCT; details in Supplementary Methods). Fovea-centered macular volume scans were performed in a 6-mm^2^ area that included the standard 1-, 3-, and 6-mm grid template from the ETDRS. Inner limiting membrane and Bruch membrane were automatically segmented by the manufacturer software (Heyex version 1.9.14.0; Heidelberg Engineering) or adjusted manually as needed by a trained ophthalmologist (M.D.V.).

**FIGURE 1:**
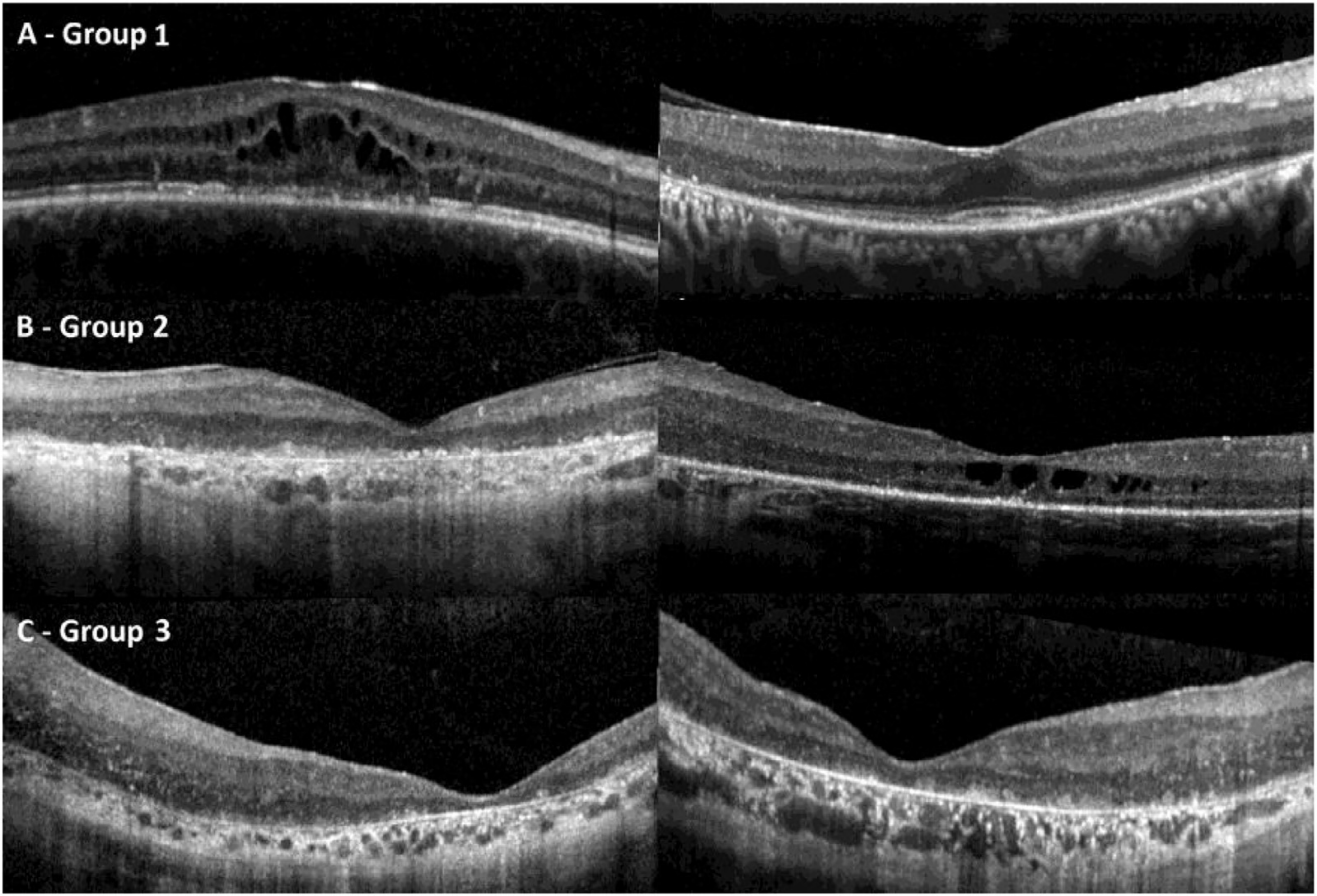
Macular optical coherence tomography findings and qualitative classification. A. Group 1, characterized by normal lamination. To the left the image corresponds to a 16-year-old patient with MD and macular cystic spaces, and to the right to a 33-year-old patient from the RP subgroup. B. Group 2, where the retinal layers are still discernible but appear ill-defined. On the left, the image corresponds to a 50-year-old individual with MD, and on the right an 11-year-old girl with LCA and macular cystic spaces. C. Group 3, defined by a disorganized retina with coalescent layers (particularly within the inner retina) and increased reflectivity of the nuclear layers. The images correspond to 2 patients with EOSRD/LCA, at age 27 years to the left and 29 years to the right. EOSRD = early-onset severe retinal dystrophy, LCA = Leber congenital amaurosis, MD = macular dystrophy.

Macular OCT scans were divided into 3 categories regarding their qualitative features, and associations were subsequently explored: group 1, characterized by normal lamination; group 2, where the retinal layers are generally discernible but appear ill-defined; and group 3, defined by a disorganized retina with coalescent layers (particularly within the inner retina) and a degree of increased reflectivity of the nuclear layers (Figure 1).^36^^,^^37^

To allow direct comparison between normal and abnormal retinal architecture, we also compared normal (group 1) and abnormal OCT lamination (groups 2 and 3). Patients with macular cysts, edema, and/or only line scans due to poor fixation were excluded from quantitative assessments (details in Supplementary Methods). Normative data regarding OCT thickness in the general population was extracted from Grover and associates,^38^ and volume parameters were taken from Roshandel and associates.^10^ Both eyes from each patient were analyzed.

### ELECTROPHYSIOLOGICAL ASSESSMENT

Pattern and full-field electroretinogram (PERG; ff-ERG) testing was performed in a subset of patients, incorporating the standards of the International Society for Clinical Electrophysiology of Vision (ISCEV) and including additional dark-adapted (DA) red flash ERGs.^39^, ^40^, ^41^ The PERG and ff-ERG were performed using gold foil electrodes, except in 9 young children who underwent testing with lower eyelid skin electrodes using a shorter ERG protocol.^42^ The quantitative ERG data analysis was limited to recordings from Moorfields Eye Hospital, to optimize consistency of methods and to minimize variability due to different types of recording electrodes. The patient ERG data were compared with those from a control group of healthy subjects (age range: 10-79 years).^43^ Further details can be found in the Supplementary Methods.

### STATISTICS

Statistical analysis was carried out with GraphPad Prism 8.0.2 (GraphPad Software). The threshold of significance for all statistical tests was set at *P* <.05. Linear regressions and *t* test were used for parametric variables’ assessment. Welch *t*-test variation was employed when the sample sizes were significantly different. χ^2^ was undertaken to assess possible association between 2 categorical variables.

## RESULTS

### DEMOGRAPHICS, PHENOTYPIC CATEGORY, AND VISUAL ACUITY

One hundred four patients (91 probands) with multiple *CRB1* variants were included in this cohort and ascertained for phenotyping, after a multidisciplinary team of ophthalmologists and clinical geneticists excluded other possible genotypes. Eighty-seven patients (84%) were seen at Moorfields Eye Hospital and 17 (16%) at St John of Jerusalem Eye Hospital. Seven additional patients had a typical phenotype for the disease (nummular pigmented deposits, admixed with small yellow or white dots, preserved PPRPE, abnormally laminated and thickened retina); however, a single disease-causing variant in *CRB1* was identified. These 7 cases with presumed *CRB1*-associated disease were not included in the analysis. Supplementary Table S1 summarizes the genetics and the clinical phenotype of these patients.

Sixty-two individuals were male (60%) and 42 female (40%). The mean age ± SD at the first visit was 19.8 ± 16.1 years, with a median of 15 years. Fifty-three participants had their first visit as children (age <16 years). The mean follow-up time of the cohort was 9.6 ± 10 years, and the overall age at their latest visit was 29.6 ± 17.2 years. The clinical findings from the cohort are summarized in Table 1.

**TABLE 1:** Clinical Characteristics of *CRB1* Disease.

Characteristic	EOSRD/LCA (n=54; 52%)	RP (n= 26; 25%)	MD (n= 24; 23%)
Age at baseline, y, mean ± SD	16.2 ± 15.3	23.9 ± 18.6	23.2 ± 13.7
Gender, n (%)			
Male	30 (56)	16 (62)	16 (67)
Female	24 (44)	10 (38)	8 (33)
Age of onset, y, mean ± SD	2 ± 2.2	13.7 ± 10.5	16.3 ± 10.8
Infancy (birth–2 y old)	35(65)	0	0
Childhood (3-11 y old)	19(35)	13(50)	9(37)
Adolescence (12-16 y old)	0	4(15)	6(25)
Adulthood (>16 y old)	0	9(35)	9(37)
Baseline best-corrected visual acuity, logMAR, mean ± SD	1.6 ± 0.8	0.9 ± 0.8	0.6 ± 0.4
Final best-corrected visual acuity, logMAR, mean ± SD	1.9 ± 0.7	1.3 ± 1	0.8 ± 0.5
Baseline WHO visual impairment category, n= (%)*			
No or mild impairment	4 (9)	10 (40)	14 (58)
Moderate impairment	15 (32)	10 (40)	10 (42)
Severe impairment	8 (17)	0	0
Blindness	20 (43)	5 (20)	0
Spherical equivalent, mean ± SD	+5.75 ± +3.5	+1.75 ± +1.75	+0.75 ± +2.5
High hyperopia	21 (39)	1 (4)	1 (4)
Myopia	2 (4)	1 (4)	7 (29)
Lens opacity, n (%)	17 (31)	15 (58)	2 (8)
Keratoconus, n (%)	4 (7)	1 (4)	0
Nummular pigment, n (%)	34 (63)	11 (42)	3 (12)
Macular involvement, n (%)	54 (100)	25 (96)	24 (100)
Baseline OCT categories, eyes n (%)			
Normal lamination and organization	0	7 (14)	11 (25)
Abnormal lamination	42 (55)	37 (74)	32 (73)
Disorganization	34 (45)	6 (12)	1 (2)
Yellow or white dots, n (%)	16 (30)	3 (12)	2 (8)
PPRPE, n (%)	16 (30)	10 (38)	3 (12)
Retinal telangiectasia, n (%)	7 (13)	4 (15)	0

EOSRD/LCA = early onset severe retinal dystrophy / Leber congenital amaurosis; RP = retinitis pigmentosa; MD = macular dystrophy; OCT = optical coherence tomography; PPRPE = preserved para-arteriolar retinal pigment epithelium.

⁎ Patients who do not appear in this classification correspond to 7 babies with LCA with non–ETDRS measured vision (eg, fix and follow) and 1 patient with RP with no details of acuity.

Based on ophthalmic history, imaging, and clinical examination, 54 individuals presented with EOSRD/LCA (52%), 26 were diagnosed with RP (25%), and 24 with MD (23%). Within the EOSRD/LCA subcohort, the mean age of onset was 2 ± 2.2 years (median 1), with poor central vision and secondly nystagmus as the most prevalent initial symptoms and signs. Among the RP group, the mean age of onset was 13.7 ± 10.5 years (median 10), and the most common presenting symptom was nyctalopia, followed by constricted field. Lastly, the patients from the MD subcohort had a mean age of onset of 16.3 ± 10.8 years (median 15) and primarily complained of decreased acuity.

Baseline BCVA was 1.6 ± 0.8 logMAR in those with EOSRD/LCA (mean age 16.2 ± 15.3 years), 0.9 ± 0.8 logMAR in patients with RP (mean age 23.9 ± 18.6 years), and 0.6 ± 0.4 logMAR in patients with MD (23.2 ± 13.7 years). Seven infants with EOSRD/LCA did not have accurate vision recorded (eg, fix and follow), and 1 patient with RP did not have BCVA detailed in the medical records. The number of patients in each WHO category of visual impairment is displayed in Table 1 and Supplementary Figure S1, A. Asymmetric BCVA was seen in 20 patients (19%): 9 with EOSRD/LCA, 5 with RP, and 6 with MD. There was a significant association between age and BCVA in all EOSRD/LCA (*P* < .0001), RP (*P* < .0001), and MD subcohorts (*P* = .047).

The mean spherical equivalent in the RP cohort was +1.75 ± +1.75 (n=1 myopic patient); in the EOSRD/LCA, +5.75 ± +3.5 (n=2 myopic patients); and +0.75 ± +2.5 among the MD subcohort (n=7 myopic patients, 29%). High hyperopia (spherical equivalent >+5.00 diopters) was found in 1 patient with RP, 1 with MD, and in 21 with EOSRD/LCA (39%).

### CLINICAL FINDINGS—ANTERIOR SEGMENT

One individual with RP and 4 with EOSRD/LCA were diagnosed with keratoconus. Fifteen patients with RP had lens opacities (58%), diagnosed at 34 ± 13 years of age; 17 patients among the EOSRD/LCA group (31%) at age 34.5 ± 14; and only 2 patients in the MD cohort, at ages 54 and 71 years, respectively. Only 4 patients among the RP subcohort had glaucoma: 1 neovascular at age 41 years, 2 open angle at ages 32 and 35 years, and 1 acute angle closure at age 29 years.

### CLINICAL FINDINGS—POSTERIOR SEGMENT

**FIGURE 2:**
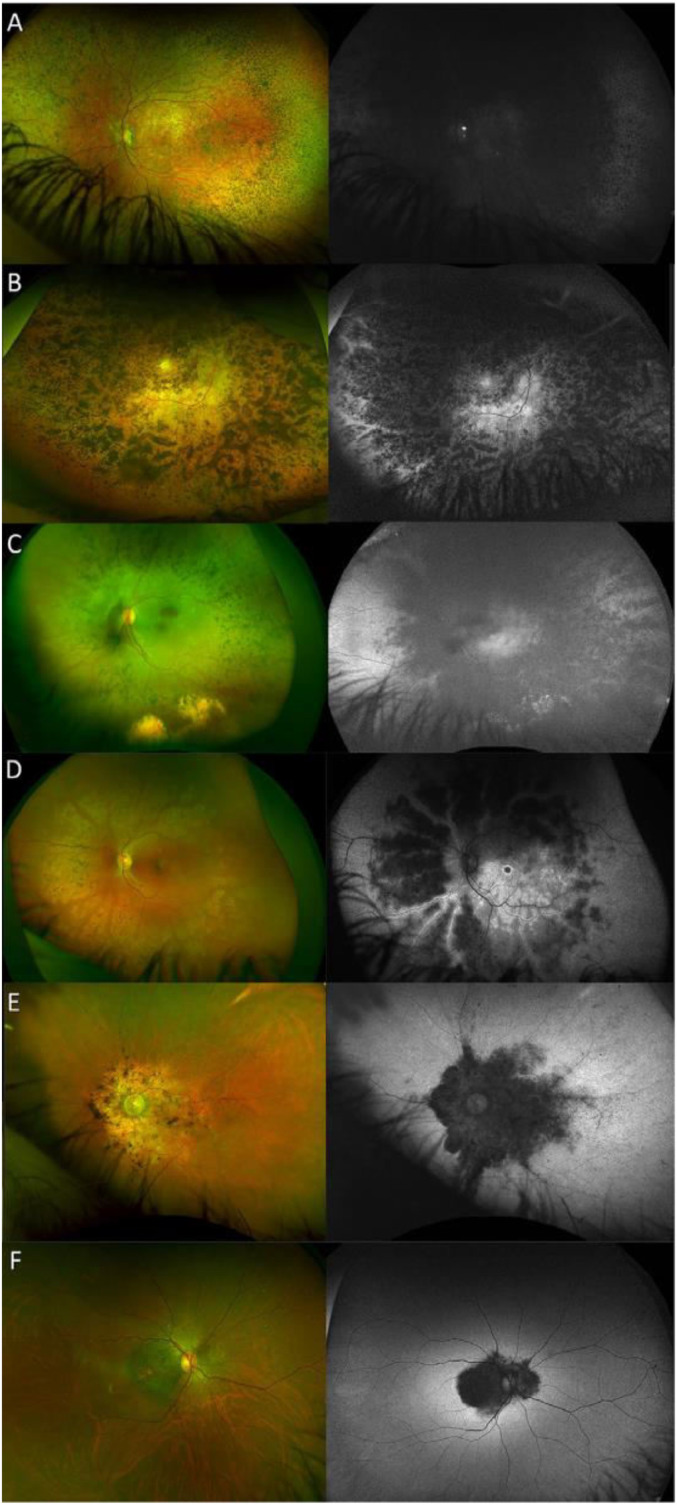
Ultrawide-field color and autofluorescence fundus images from individuals with *CRB1* retinopathy. A. Thirty-year-old patient with EOSRD/LCA. His parents and pediatrician noticed he had nystagmus as an infant and he had poor vision his entire life. Deep pigmented nummular lesions, as well as yellow/white dots mainly temporal to the macula, are seen. AF is decreased and an optic disc drusen is visible. B. Sixty-one-year-old patient with LCA. More coalescent pigment deposits and profound macular atrophy are seen. In the AF image, preserved para-arteriolar retinal pigment epithelium (PPRPE) is more readily seen. C. Twenty-eight-year-old with RP and retinal telangiectasia that resulted in exudation and vitreous hemorrhages. D. Eighteen-year-old with RP, with few pigment deposits, macular involvement, and PPRPE. E. Seventy-year-old patient with MD. He reported decreased central vision since age 15 years and normal peripheral field. F. Forty-two-year-old with MD. He had failed a vision screening test at age 24 years old, and his vision had been slowly decreasing since. Of note is the characteristic pattern of hypoautofluorescence that involves the macula and all optic disc borders. AF = autofluorescence, EOSRD = early-onset severe retinal dystrophy, LCA = Leber congenital amaurosis, MD = macular dystrophy, PPRPE = preserved para-arteriolar retinal pigment epithelium, RP = retinitis pigmentosa.

All EOSRD/LCA patients presented with diffuse, dense pigment in the retinal periphery, with both spicules and nummular pigment. They all had macular involvement, with a pigmented ring in the posterior pole in 16 patients, and coloboma-like severe atrophy in 4 (Figure 2, A and B). All patients in the RP subcohort presented with peripheral retinal pigmentation and a range of phenotypes at the posterior pole: 7 patients had a normal-appearing macula, 10 had a blunted or opaque macular reflex, and 12 had signs of atrophy and pigmented deposits (Figure 2, C and D).

Sixteen patients with MD had normal retina outside the arcades, whereas 8 had peripheral areas of pigmented bone spicules or nummular lesions, and/or vessel thinning. The macular reflex was blunted in 12 patients, with mottled retinal pigment epithelium and signs of atrophy in the remaining 12—the latter often accompanied by pigmented bone spicules and affecting the nasal peripapillary area (Figure 2, E and F). Four individuals with MD presented with foveal sparing and therefore good central vision. Macular involvement (functional and/or structural) was present in 25 RP patients (first documented at a mean age of 23.9 + 18.6 years), and in all EOSRD/LCA (2.4 ± 2.8 years) and MD patients (19 ± 10 years).

Thirty-four individuals with EOSRD/LCA (mean age 24.1 ± 16.6 years), 11 with RP (23 ± 18 years), and 3 with MD (37.3 ± 11.1 years) had nummular pigment clumps. White or yellow dots were seen in 16 EOSRD/LCA patients (mean age 24 ± 9 years), in 3 patients with RP (19 ± 7.5 years), and in 2 MD patients, at the same time point where nummular pigment was present (25 and 35 years of age). Sixteen patients with EOSRD/LCA (mean age 22 ± 13.5 years) presented with PPRPE, 10 with RP (23 ± 12 years), and 3 with MD (38 ± 10.5 years). PPRPE was more readily identified with autofluorescence (AF) imaging, likely given the retinal pigment epithelial basis of AF imaging.^10^

Seven EOSRD/LCA patients had retinal telangiectasia (mean age 30 ± 10 years), 6 of which were associated with exudation, with 2 resulting in vitreous hemorrhage(s). Retinal telangiectasia was seen in 4 patients with RP, diagnosed at a mean age of 28 ± 8 years, and resulted in a range of complications including exudation, vitreous hemorrhage, and retinal detachment (RD). Optic disc drusen was the most common optic nerve abnormality, affecting 7 patients with LCA, 4 with RP, and 1 with MD. Two patients with EOSRD/LCA had gliosis on the optic disc.

Ocular complications were seen in 12 individuals with EOSRD/LCA (21%); in 4 they were related to telangiectasia, whereas the remaining 8 corresponded to RD, iris nodules, anterior segment synechiae, retinal hamartoma, and corneal hydrops. Ocular complications were also reported in 6 patients with RP (22%): 4 were associated with retinal telangiectasia, and the remaining 2 were uveitis and asteroid hyalosis. Only 1 patient with MD was found to have unusual vessel sheathing at age 8 years, of unknown cause.

### MACULAR OCT ANALYSIS

Eighty-five patients from our cohort had macular OCT scans (82%): 38 (45%) with EOSRD/LCA, 25 (29%) with RP, and 22 (26%) with MD—all being included in the qualitative analysis. Quantitative assessment was possible in 15 patients with EOSRD/LCA, 8 with RP, and 10 with MD, because of image quality and sufficient scans being available. Eleven individuals from the EOSRD/LCA subcohort had follow-up scans (over 7 ± 3 years), 7 from the RP group (over 7.5 ± 1.52 years), and 10 with MD (7 ± 3 years), which enabled additional longitudinal analysis. Baseline and follow-up structural parameters are described in Supplementary Table S2.

**FIGURE 3:**
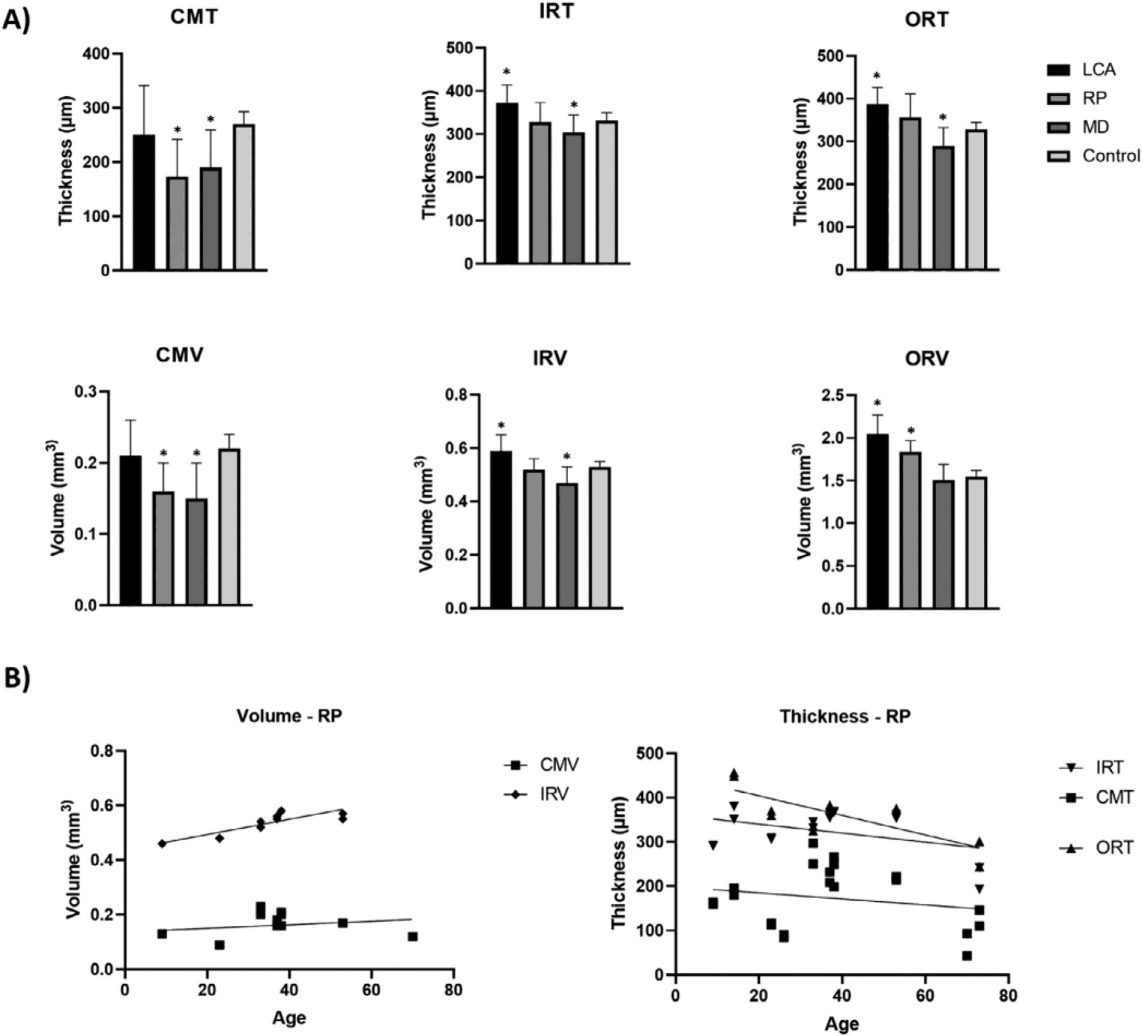
Graphic representation of statistical analysis undertaken to analyze various structural optical coherence tomography parameters. A. Bar graphs comparing structural measurements in each group to a control group. Significant differences in all groups are seen (marked with *), with the EOSRD/LCA group showing significantly increased inner ring thickness (IRT), outer ring thickness (ORT), inner ring volume (IRV), and outer ring volume (ORV); the RP group with decreased central macular thickness (CMT) and central macular volume (CMV), and increased ORV; and the patients with MD with thinned CMT, IRT, ORT, CMV, and IRV. B. Linear regression representation of the association between age and structural parameters in the RP subcohort, where we see a significant, positive slope regarding volume (IRV), and a negative one when it comes to thickness (ORT). EOSRD = early-onset severe retinal dystrophy, LCA = Leber congenital amaurosis, MD = macular dystrophy, RP = retinitis pigmentosa.

The EOSRD/LCA subcohort had significantly increased inner ring thickness (IRT, *P* = .002), outer ring thickness (ORT, *P* < .0001), inner ring volume (*P* = .002), and outer ring volume (*P* = .0004) compared with the normal population (Figure 3). The RP group had significantly decreased central macular thickness (CMT, *P* = .0008) and central macular volume (*P* = .0037); however, the outer ring volume was increased (*P* = .0004). Lastly, patients with MD showed significant thinning in all CMT (*P* = .003), IRT (*P* = .004), ORT (*P* = .01), central macular volume (*P* = .0017), and inner ring volume (*P* = .01; Figure 3, A).

No association was found between thickness or volume metrics and age in EOSRD/LCA (*P* between .08 and .95) and MD subgroups (*P* between .4 and .8). However, a significant association was found in patients with RP between age and ORT (negative slope, *P* = .003) and inner ring volume (positive slope, *P* = .0003; Figure 3, B).

Longitudinal analysis demonstrated no statistically significant differences between baseline and follow-up parameters in all subcohorts. Sixty percent of patients with EOSRD/LCA had decreased CMT over follow-up (mean –3 µm/y), 54% had decreased IRT (–1.5 µm/y), and 50% had decreased ORT (–0.6 µm/y). Within the RP subcohort, 67% had decreased CMT (–1.9 µm/y), 49% had decreased IRT (with an overall increase, however, of 0.3 µm/y), and 43% had decreased ORT (increase of 1.5 µm/y). Lastly, 59% patients with MD had decreased CMT over follow-up (–0.5 µm/y), 70% had decreased IRT (–1.1 µm/y), and 49% had decreased ORT (–1.8 µm/y).

Macular cystic spaces were present in 29% of our cohort as a whole, and in 52% and 42% of the RP and MD subgroups, respectively. The majority had only the internal nuclear layer (INL) affected (24 eyes), followed by both INL and outer nuclear layer (ONL) involved (19 eyes), and then ONL only (13 eyes). There was a significant difference in the age of patients with and without cysts, both analyzing the cohort as a whole (*P* = .007) and excluding the EOSRD/LCA group (*P* = .002), with a mean age of 22.3 ± 15.4 years for those having cysts and 34.6 ± 18.2 for those without. The cysts resolved during follow-up in 24% of eyes, without any treatment. The presence of outer layers at the fovea and perifovea was also recorded (Supplementary Results).

In terms of qualitative assessment, overall, 8 patients had both eyes in OCT group 1 (9%, 28.9 ± 16.4 years old), 52 in group 2 (61%, 25.2 ± 15.4 years old), and 18 in group 3 (21%, 43.2 ± 14.8 years of age, 16 of which were in the EOSRD/LCA cohort; Figure 1 and Supplementary Figure 1, B). Two patients had one eye in group 1 and the other in group 2, and 5 patients had one eye in group 2 and the other in group 3. Considering the cohort as a whole, there was a significant age difference between groups 1 and 3 (*P* = .03) and 2 and 3 (*P* = .0003), whereas no age difference was recorded between groups 1 and 2 (*P* = .49). Visual acuity was also significantly different between groups; 0.4 ± 0.32 logMAR in group 1, 1.25 ± 0.97 logMAR in group 2, and 3.27 ± 1.38 logMAR in group 3, with progressive VA decline as the OCT layers became more disorganized (*P* < .0001).

Within the EOSRD/LCA group, 20 patients had both eyes in OCT group 2 (53%, age 19.6 ± 11.1 years), 16 in OCT group 3 (42%, age 38.4 ± 16.9 years), and 2 each eye in groups 2 and 3 (ages 15 and 27 years). The RP subcohort had 3 patients in group 1 (12%, 34 ± 14 years old), 17 in group 2 (68%, 22.8 ± 17.9 years old), 2 in group 3 (44 and 53 years old), 1 with one eye in group 1 and the other in group 2 (37 years old), and 2 with one eye in group 2 and the other in group 3 (38 and 73 years old, respectively).

Lastly, the MD group had 5 patients in group 1 (23%, 16.3 ± 17 years old), 15 in group 2 (68%, 31.7 ± 15.8 years old), 1 with one eye in group 1 and the other in group 2 (15 years old), and 1 with one eye in group 2 and the other in group 3 (23 years old). Findings are summarized in Table 1. Focusing on the EOSRD/LCA subcohort, the age difference between groups 2 and 3 was significant (*P* = .0005), whereas no age difference was found between groups 1 and 2 of the RP (*P* = .28) and MD subcohorts (*P* = .07).

In addition, given image quality variation, potential challenges to robustly distinguish between OCT groups 2 and 3, and the desire to compare definitively normal with abnormal architecture, we also compared group 1 (normal lamination) vs groups 2 and 3 combined (abnormal lamination). The latter (group 2+3) had a mean age of 26.9 ± 16.8 years, and there was no significant age difference when compared to the normal lamination group (*P* = .81). The visual acuity of the abnormally laminated group was 1.75 ± 1.39 logMAR, which was significantly different from group 1 (*P* < .0001).

In terms of transition between groups over time, 4 eyes (3 patients) with EOSRD/LCA changed from group 2 to 3, whereas 5 (3 patients) with RP and 5 (3 patients) with MD went from group 1 to group 2 during follow-up (further details in Supplementary Results).

### ELECTROPHYSIOLOGY

Twenty-nine patients (28%) underwent ff-ERG and PERG. There was a high degree of inter-ocular ERG symmetry based on amplitudes of the DA 0.01, DA 3, and DA 10 ERG a- and b-waves, LA 30-Hz ERG and LA 3 (single flash) ERG b-waves (slope = 1.00; *r*^2^ = 0.98), and on the peak times of the DA 10 ERG a- and b-waves and LA 30-Hz ERGs (slope = 0.98; *r*^2^ = 0.96). PERG N95 data were available for analysis in 12 of 14 patients with a detectable P50 component. The N95:P50 ratio had a mean value of 1.7 (range 1.4-3.0), which is similar to the reference group for the lab (mean = 1.5)^44^; that is, there was no evidence of N95 reduction disproportionate to P50 in this small group.

**FIGURE 4:**
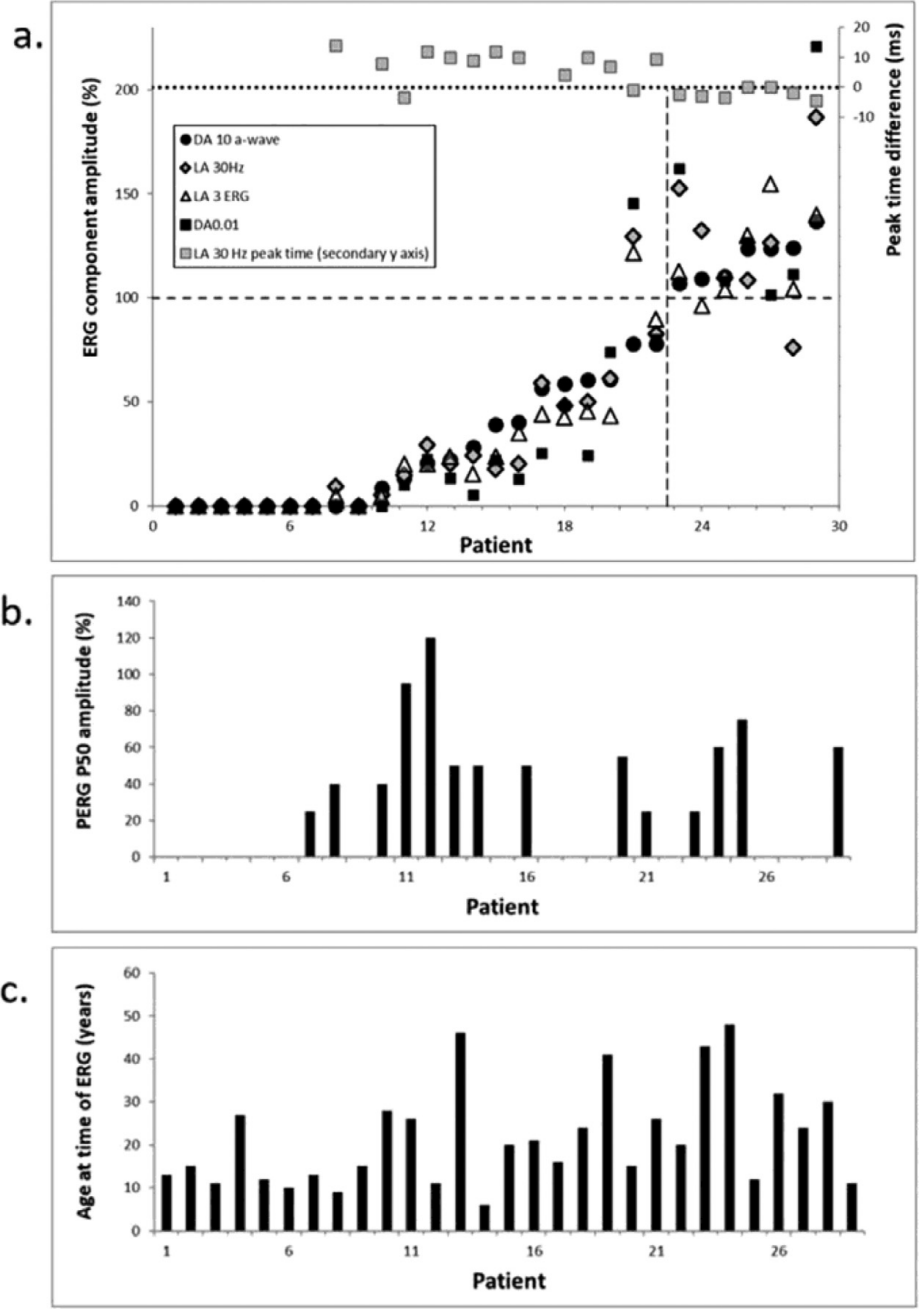
Quantification of the full-field ERG and PERG findings. Full-field ERG findings summarized in 29 subjects tested according to the ISCEV standard methods. A. The amplitudes of the DA 10 ERG a-wave, LA 30 Hz ERG, and LA 3 ERG b-wave are plotted against the primary axis as a percentage of the age-matched lower limit of the (“normal”) reference range, with values arranged in ascending order of DA 10 ERG a-wave amplitude for clarity. The LA 30-Hz peak times are plotted as a difference from the age-matched upper limit of normal timing against the secondary axis. B. PERG P50 amplitudes plotted as a percentage of the lower limit of normal amplitude for the same order of subjects as in panel A; the absence of a column indicates undetectable responses. C. The age of the patients at the time of testing, arranged in same order as in panels A and B. Note the 7 subjects to the right of the vertical broken line in panel A had normal ERGs but abnormal PERG P50 components (B), consistent with a diagnosis of macular dystrophy. The LA 30Hz ERG in subject 29 was subnormal, but in presence of significant eye closure. DA = dark-adapted, ERG = electroretinogram, PERG = pattern electroretinogram.

**FIGURE 5:**
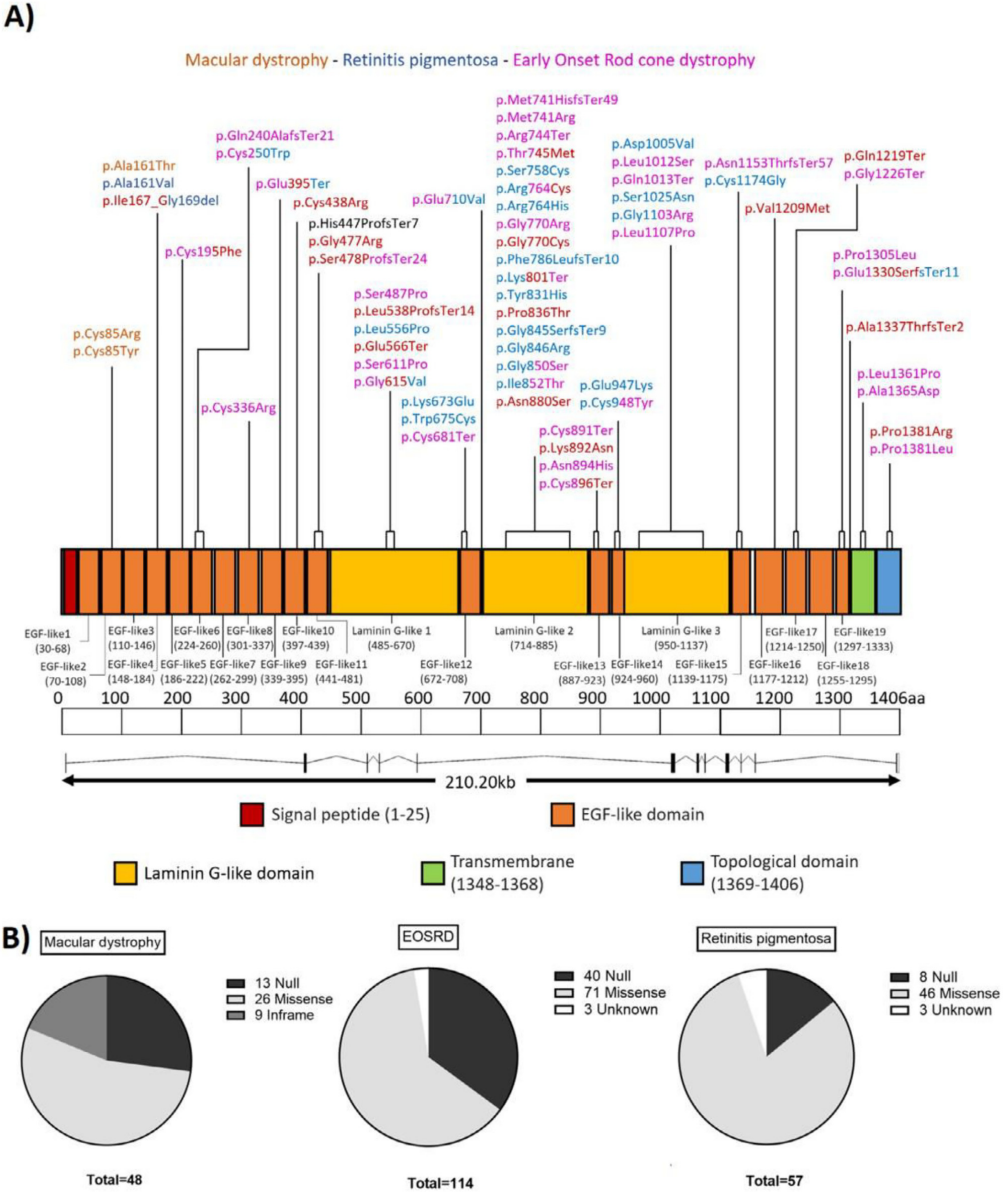
A. Graphic representation of the *CRB1* gene and protein, with details on functional domains. Each variant within our cohort is detailed above the protein, with its corresponding location, and with different colors according to the associated phenotype. B. Pie graph representation of the type of variants seen in each phenotypic group. The larger prevalence of null changes can be easily visualized in the MD and EOSRD/LCA groups, compared with the RP group, and also the high prevalence of the p.Ile167_Gly169del in-frame deletion, found exclusively in the MD group. EOSRD = early-onset severe retinal dystrophy, LCA = Leber congenital amaurosis, MD = macular dystrophy, RP = retinitis pigmentosa.

Figure 4 summarizes the electrophysiological findings, and Supplementary Figure S2 shows representative recordings. Eight patients with a clinical diagnosis of EOSRD/LCA aged between 9 and 27 years had undetectable ERGs under all stimulus conditions. The ERGs in others (8 patients with MD and 6 with RP) revealed a similar degree of rod and cone system involvement, although there was evidence of slightly greater rod than cone system dysfunction in 5 cases, revealed by proportionately greatest reduction in the rod-system selective DA 0.01 ERG (Figure 5, A; patients 8, 13, 14, 16, and 19). Seven patients had MD both clinically and electrophysiologically, including 6 with normal ERGs and 1 with mild reduction in the LA 30 Hz ERG (Figure 4, A; patient 28), but likely due to eye closure noted during testing. Further details are described in the Supplementary Results and Supplementary Figures S2 and S3.

### LONGITUDINAL ANALYSIS

Follow-up BCVA was obtained in 41 (76%) individuals with EOSRD/LCA, 20 (77%) with RP and 20 (83%) with MD. Mean follow-up time was 9.9 ± 12.1 years for patients with EOSRD/LCA, 9.9 ± 9.8 for the RP subcohort, and 7.6 ± 5.7 for MD patients. Follow-up BCVA was 1.9 ± 0.7 logMAR in patients with EOSRD/LCA (mean age 27 ± 17 years), 1.3 ± 1 logMAR in patients with RP (mean age 38 ± 13 years), and 0.8 ± 0.5 logMAR in MD patients (mean age 30 ± 16). There was a significant difference between baseline and follow-up BCVA in the EOSRD/LCA (*P* < .0001), RP (*P* < .0001), and MD (*P* = .016) subcohorts.

The rate of BCVA decline was 0.06 logMAR (3 letters)/year in patients with EOSRD/LCA, 0.07 logMAR (3.5 letters)/year in patients with RP, and 0.04 logMAR (2 letters)/year in patients with MD. Fifteen patients (37%) with EOSRD/LCA dropped 15 ETDRS letters or more over follow-up in at least 1 eye, 10 (50%) with RP, and 7 (35%) with MD. Fourteen patients (34%) with EOSRD/LCA progressed to more advanced WHO categories of visual impairment, 7 (35%) with RP, and 6 (30%) with MD. Overall, 20 patients (25%) became severely sight impaired and/or blind over follow-up: 12 with EOSRD/LCA, 5 with RP, and 3 with MD.

Macular OCT longitudinal evaluation demonstrated that EOSRD/LCA patients may experience slowly decreasing retinal thickness (despite their baseline thickening), progressive loss of lamination, and degeneration of both central and pericentral outer retinal layers over follow-up. *CRB1*-RP patients in our sample experienced loss of thickness at the fovea and parafovea, while increasing in the perifovea; however, the small sample size available for longitudinal assessment should be borne in mind. MD patients had progressive thinning of the posterior pole—associated with poor retinal lamination—and discontinuous/narrower outer layers.

### GENETICS

Seventy-eight different *CRB1* variants were found in our cohort, 46 of which were missense (59%), 11 frameshift (14%), 10 nonsense (13%), 5 canonical splice site alteration (6%), 2 missense with significant splice site alteration (3%), 2 intronic variants (3%), 1 in-frame deletion (1%), and 1 deletion of exon 12 (1%) (Supplementary Table S3). Schematic representation of these detected variants and the status of evolutionary conservation are presented (Figure 5, A; Supplementary Figures S4 and S5). Twenty-eight variants were classified as pathogenic (36%), 43 (55%) were likely pathogenic, and 7 (9%) were variants of uncertain significance. Twenty-three variants (29%) were novel, whereas 55 were previously reported. c.2843G>A, p.(Cys948Tyr) was the most common variant, seen in 15 individuals (14 probands), 14 of which had EOSRD/LCA. This was followed by c.498_506delAATTGATGG, p.(Ile167_Gly169del), found in 9 probands with MD. Another frequent variant among the MD group was p.Pro836Thr, present in 7 Black African individuals (4 families).

Null variants (including nonsense, frameshift, splicing, and exon deletion) were significantly more associated with both EOSRD/LCA (*P* = .001) and MD (*P* = .003) phenotypes, rather than with RP (Figure 5, B). Double null genotypes were only seen in EOSRD/LCA (12 patients). The 4 variants p.Cys896Ter, p.Thr745Met, p.Ser478ProfsTer24, and p.Cys195Phe and the 3 loci p.Gly770, p.Gln1219, and p.Pro1381 harbored changes causing both EOSRD/LCA and MD (Figure 4, A). In the MD cases, however, the aforementioned variants and loci were combined either with p.(Ile167_Gly169del), previously characterized as a hypomorphic allele,^8^ or with a likely pathogenic variant. Eight individuals with MD (33%, 7 probands) did not harbor the in-frame or the p.Pro836Thr variants. p.Asn880Ser was the only repeated variant among the latter, seen in 2 unrelated families.

Intrafamilial variability was present in 2 families. In one family, a sibling had mild, asymptomatic MD and the other had RP with decreased vision since her 20s (homozygous p.Ser638Leu). In the other family, 3 different *CRB1* variants were found to be segregating; a great uncle inherited p.Cys195Phe and p.(Ser478ProfsTer24) and developed EOSRD/LCA, and 1 nephew harbored p.(Ser478ProfsTer24) and the in-frame p.(IIe167_Gly169del) and presented with MD.

## DISCUSSION

This study characterizes the largest cohort of patients with molecularly confirmed *CRB1* retinopathy as of this writing, with 23 novel *CRB1* variants identified. Deep phenotyping data are presented, including detailed analyses of demographic features, fundus imaging, morphologic characteristics, and quantitative data of electrophysiological assessment, with the aim of establishing clear phenotypes, potential outcome measures, genotype-phenotype correlations, and laying the foundations for future studies that aim to develop therapies and treatment strategies.

Low vision was the most common WHO category for individuals with EOSRD/LCA in the first 2 decades of life, followed by blindness from 20 years on. In the RP group, there was severe visual impairment from age 40 years, whereas in the MD group it was only seen in a minority of cases from age 28 years. This is similar to the observations from Talib and associates,^45^ who reported blindness among *CRB1*-RP patients from the fifth decade of life. Patients with MD appeared to have an overall good prognosis, with relatively preserved visual function until adult age in at least 1 eye. The findings highlight the window of opportunity for potential treatments such as gene therapy.

Individuals with EOSRD/LCA may need relatively early intervention in childhood and adolescence because of early macular structural and functional involvement, whereas patients with RP or MD would potentially benefit up until their late 30s. The latter is in agreement with Talib and associates^46^ and Mathijssen and associates,^20^ whereas Nguyen and associates^37^ recommended treatment before the third decade of life, having seen in their cohort a faster decline around that period. Furthermore, in our cohort, VA deterioration was significantly associated with age in all EOSRD/LCA, RP, and MD subcohorts. This association was also found in *CRB1*-RP by Mathijssen and associates,^20^ but not by Talib and associates,^36^ despite their cohort being mostly RP phenotype.

Employing their statistical approach of age vs BCVA of the best-seeing eye (instead of age vs BCVA of both eyes as we have herein), we still obtained a significant association (*P* = .001). Regarding BCVA asymmetry, we report a smaller proportion than previous cohorts (19% in this report vs 33% and 31% in other groups).^45^^,^^46^ Progressive visual decline was also reported during long follow-up studies^20^; however, this was not picked up in the 2-year follow-up study by Nguyen and associates.^37^ The rate of progression of our patients was greater than the one reported by Talib and associates,^45^ possibly reflecting an overall earlier onset and more severe disease stage in our patients.

The median age of onset in the RP subgroup was 10 years, which was higher than the Dutch and Belgian cohorts (4 and 5 years each).^45^^,^^46^ Therefore, 45% of our RP group had a disease onset after the first decade of life (vs 15% and 20% in the aforementioned cohorts). We appear to have captured a subset of individuals with mild, adult-onset disease, previously poorly characterized. Seven of these patients (14-53 years of age) had preserved foveal architecture and good central vision at their latest visit.

Hyperopia was the prevalent refractive error in all *CRB1* phenotypes, with 39% of the EOSRD/LCA patients having high hyperopia (>+5.00 D). Wang and associates^47^ found >80% of their *CRB1*-LCA cohort to be highly far-sighted, and Mathijssen and associates^20^ also found consistent hyperopia in their *CRB1*-RP cohort. This was not, however, a pathognomonic feature in *CRB1*-MD (29% of our patients were myopic and 16% were emmetropic), corroborating a previous study.^45^

The most prevalent posterior segment feature within our cohort was maculopathy, which was present in 97% of our cohort, with variable severity, and consistent with smaller studies.^45^^,^^47^ Other common features included nummular pigment, PPRPE, white or yellow dots, and retinal telangiectasia. We did not find significant differences between the ages at which white or yellow dots and nummular pigment were found, and sometimes both appeared simultaneously (Figure 2, A); therefore, it is unclear if these represent different stages of the same lesion.^20^^,^^47^ Of note, pigmented paravenous chorioretinal atrophy, previously associated with *CRB1* on one occassion,^48^ was not seen in any of our genetically confirmed patients.

Macular OCT revealed a range of features; 65% of the eyes from our cohort had ill-defined lamination (group 2; Figure 1), and 24% had disorganized retinal layers (group 3). Previous studies have reported similar values, although these may depend on the cohort characteristics; for example, EOSRD/LCA patients have a more severe phenotype. Our results are comparable with some previous investigations, for example, where 38% to 52% eyes were categorized as group 3.^37^^,^^45^ However, we consider that the classification of OCTs into groups 2 and 3 can be somewhat subjective, and it is also dependent on the quality of the scan, intrinsically difficult in patients with EOSRD/LCA and nystagmus.

To mitigate this potential limitation and thereby minimize possible bias, we added the comparison of normal and abnormal lamination groups. Patients in group 3 were older and our longitudinal data (available in 7% of eyes) showed progressive loss of defined lamination on qualitative review; therefore, the poorly laminated and disorganized OCT in *CRB1* may also involve a degenerative process and not only a congenital one. This is consistent with previous suggestions, although far larger studies with greater serial data are required.^46^ However, it is of note that an age difference was not present when comparing the groups of normal vs abnormal OCT lamination. Nevertheless, given its association with poor visual function, OCT layer organization should be considered as a criterion to take into account for future treatment trials.^36^^,^^37^ Degeneration of central and pericentral outer layers during follow-up was also reported by other groups in longitudinal analysis.^10^

Quantitative analyses of OCT have revealed a strong association between CRB1 and a thickened retina, reported in animal models^49^ as well as in up to 82% of the individuals from a previous cohort.^46^ The perifovea (outer ETDRS ring) has been the most commonly described area with increased thickness, with limited evidence regarding the central and inner ring areas.^10^^,^^14^^,^^20^^,^^46^ We observed that our EOSRD/LCA and RP groups had significantly increased ORT, when compared to the reference population. We also noticed increased inner ring values in our EOSRD/LCA cohort exclusively, previously unreported, whereas patients with *CRB1*-MD seemed to be exempt from the retinal thickening characteristic.

Some groups have also reported age-dependent posterior pole thinning.^10^^,^^20^ Although we found decreased macular thickness longitudinally in our EOSRD/LCA and MD subcohorts, this did not reach statistical significance. We found a significant decrease in ORT with age, concomitantly with increased inner ring volume in the RP subgroup. It is possible that the thinning follows a centripetal pattern and the parafovea remains preserved until later stages, yet a larger number of patients are needed to arrive at a more definitive conclusion. The inner retina is generally where the increased thickness is most noticeable, which may be due to excess ganglion cells.^14^ Our hypothesis is that increased retinal thickness is due to *CRB1*-related poor adhesion between cells, which leads to poor lamination and abnormal anatomy, consequently occupying volume in a less efficient, broader way.^50^^,^^51^

Macular cystic spaces were present in a lower percentage in our cohort when compared to other groups, and this may be related to the higher proportion of EOSRD/LCA patients, who are rarely affected by macular fluid accumulation.^36^^,^^45^^,^^46^ We were able to associate younger age with the presence of macular cystic spaces, a relationship that was previously hypothesized.^36^ Cysts resolved in nearly a quarter of the eyes during follow-up without any treatment, which is noteworthy given their reported poor responsiveness to treatment.^52^^,^^53^ We can conclude that cystoid maculopathy is a prevalent feature in *CRB1*-RP and -MD, affecting mostly younger individuals, and it can be self-limiting. This has caused *CRB1* to be included within the differential diagnoses considered in cystic maculopathies, such as those secondary to variants in *RS1* and *NR2E3*.^54^

Although macular fluid accumulation is also seen in other RP genotypes,^55^ the pathophysiology may be easier to explain in *CRB1* retinopathy. *CRB1* transcript CRB1-A has been located within Müller glia cells in mouse and human retina,^25^^,^^56^ its loss manifesting as an irregular number and size of their apical villi.^57^ Müller cell loss of integrity may lead to abnormal fluid transport and has been associated with macular edema^58^; hence, this may be the cause of the cystic spaces found in some *CRB1* phenotypes.

The variant c.2843G>A has been reported the most commonly occurring in individuals with *CRB1*-related disease, having a carrier frequency of approximately 2:10 000.^59^ c.2843G>A, p.(Cys948Tyr) was also the most prevalent in our cohort, as well as in other European groups.^4^^,^^60^ However, in a Dutch cohort p.(Met1041Thr) was the most prevalent,^37^ and in a Chinese cohort p.(Gly1226Ter) was most common, and the c.2843G>A allele was not identified.^47^

The in-frame deletion p.(Ile167_Gly169del) was reported to be the most prevalent disease-causing *CRB1* variant in non-Asians,^8^ particularly frequent in Spanish patients.^60^ It was previously described as a hypomorphic allele, which led to limited retinal dysfunction.^8^ However, 2 Spanish reports related it to early-onset RP and LCA, without further phenotypic details.^60^^,^^61^ We found p.(Ile167_Gly169del) in 9 patients with MD. Eighty percent of these patients had cystic macular changes, and 90% did not have visual impairment as per WHO classification. Therefore, this variant may be associated with a good clinical prognosis,^8^ with possibly limited retinal involvement (yet not necessarily macula restricted).^45^

Another frequent variant among the MD group was p.(Pro836Thr), present in 7 Black African individuals (4 families). This variant was first described by den Hollander and associates, seen in a patient with RP and PPRPE, without a known second variant, and with no details regarding race.^16^ It was also reported in 2 identical twin sisters with cystic maculopathy.^62^ It has a frequency of 0.00277 and 0.0028 in the Black African population or descendants (an allele frequency of exome and genome: gnomAD v2.1.1), and it currently has conflicting evidence regarding its pathogenicity (ClinVar ID: 372352). The new cases associating it with disease, both in compound heterozygous and homozygous state, contribute toward classifying it as disease-causing.

Lastly, we were able to test the association of null variants and different phenotypes. We not only found them significantly associated to EOSRD/LCA, as previously suggested by other groups,^15^^,^^45^^,^^63^ but also to MD, representing 36% and 26% of the variants seen in each group. In contrast, however, null variants in the MD subcohort were combined with likely hypomorphic and milder pathogenic variants, thereby decreasing the overall functional impact of these genotypes. Also, the relatively small number of MD patients compared with EOSRD/LCA patients in our cohort needs to be borne in mind.

The large size of *CRB1*, occupying nearly all the AAV packing capacity, has made the development of a therapy for these retinopathies challenging.^64^ However, novel approaches through AAV vectors with small promoters have allowed the expression of CRB1 protein in Müller glial cells in vitro.^65^ Another option tested in vivo was the supplementation of CRB2, a smaller yet highly similar protein in structure, that improved the photoreceptors’ morphology and function after delivery.^66^ These creative approaches, as well as the innovative human induced pluripotent stem cells (iPSC) disease models, will facilitate progress to developing treatment options.^67^ Furthermore, the detailed characterization of *CRB1* macular OCT features will likely be helpful toward its inclusion among the inherited retinal dystrophy genotypes that are currently used to train and validate deep learning systems for automated diagnosis and classification.^68^

Inherent limitations of our study are its retrospective design, the differences in follow-up time between patients, not all data being available for every individual, and different types of accessible data, acquired with diverse methods and protocols. The above-mentioned limitations were ameliorated by the size of our genetically confirmed cohort, the wide age range of our patients, the international representation, and their detailed medical records.

In conclusion, this multinational investigation characterizes the largest cohort of patients with molecularly confirmed *CRB1* retinopathy as of this writing, identifies novel *CRB1* variants, and delineates genotype-phenotype correlations. Clinical and functional phenotypes are detailed, establishing the range and combinations of features that will aid diagnosis and inform patient management. Potential endpoints for future natural history and interventional clinical trials are highlighted, laying the foundations for future studies that aim to develop therapies and treatment strategies.

## Appendix Supplementary materials

Image, application 1

Image, application 2

Image, application 3

Image, application 4

Image, application 5

Image, application 6

Image, application 7

Image, application 8

Image, application 9
