# Supplementary material for: CRB1-Associated Retinal Dystrophies: Genetics, Clinical Characteristics, and Natural History
Source: Am J Ophthalmol. 2023 Feb;246:107–21. doi: 10.1016/j.ajo.2022.09.002 (PMC10555856; doi:10.1016/j.ajo.2022.09.002)
Supplement: Supplementary file 1 [file mmc1.doc]

**Supplementary Methods**

Imaging. Color and autofluorescence retinal imaging were undertaken with Optos Panoramic 200 ultrawide-field retinal imaging device (Optos PLC, Dunfermline, Scotland), Topcon (Topcon Corp., Tokyo, Japan), and Zeiss Clarus (Carl Zeiss Meditec Inc., California, USA). Near-infrared reflectance and OCT imaging were acquired with Spectralis SD-OCT device (Heidelberg Engineering, Heidelberg, Germany) and OCT Triton (Topcon).

*In silico* molecular genetic analyses. All recruited patients were reassessed for their detected *CRB1* variants. Sequence variant nomenclature was obtained according to the guidelines of the Human Genome Variation Society (HGVS) by using Mutalyzer. Classification of all detected variants was also performed mainly based on the guidelines of the American College of Medical Genetics and Genomics (ACMG). An additional classification was applied for the evaluation of the verdict assessment and PM2 and PP4. Minor allele frequency for the identified variants in the general population was assessed in the Genome Aggregation Database (gnomAD) datasets. The population data and general coverage were also provided with the gnomAD database. General prediction scores were further calculated using MutationTaster, FATHMM, REVEL, and the Combined Annotation Dependent Depletion (CADD). Functional prediction was performed employing SIFT, PROVEAN, and Polyphen 2. Human splicing finder 3.0 was applied for splicing defects prediction. Mammalian (PhyloP30way and PhastCons30way) and vertebrate (PhyloP100way and PhastCons100way) conservation were also investigated. The previously reported variants were surveyed with the HGMD database and ClinVar database (accessed on 1^st^ November 2021).

Electrophysiology. Pattern ERG P50 was used to assess macular function, and the full-field ERG to assess generalised (mainly peripheral) rod and cone system function. Pattern ERGs in the same young cases were recorded to a standard stimulus field size (15 degrees by 12 degrees) and/or to a large stimulus field (30 degrees by 24 degrees).

The amplitudes of the main full-field ERG components were plotted as a percentage of the age-matched lower limit of “normal” or as a difference from the upper peak time limit, including the dark adapted (DA) 10 ERG a-wave, and the light-adapted (LA) 3 single flash ERG b-wave and the LA 3 30Hz ERG. To take into account non-Gaussian distribution within the control group, the limits were defined as the lowest amplitude value in the control group minus 5% of the reference range (maximum minus minimum values) for amplitudes or plus 5% of the reference range for peak times.^1,2^

**Supplementary Results**

Macular OCT analysis. Within the EOSRD/LCA group, eleven patients had a macular coloboma-like configuration (only noticeable by fundoscopy in 4). Three patients had eyes that changed from group II to III during follow up, two of them just one eye (over a period of 3 and 8 years each) and one both eyes (follow up time 8 years). Four individuals had discontinuous yet present outer layers subfoveally on both eyes, and two in one eye only (age range 9 – 31 years). Perifoveal outer layers were found in 5 patients in both eyes and in 2 patients unilaterally (age 9 – 27 years of age). Central and pericentral outer layers were lost during follow up in 4 eyes each (8 – 12 years follow up). Three patients (6%, ages 8, 9 and 28 years) had cystic macular spaces at the outer nuclear layer (ONL) in both eyes at baseline, with only one eye persisting after 12 years of follow up.

The RP group had volume scans in 18 patients and line scans only in 7 patients. During follow up (3 – 6 years), 5 eyes went from group I to group II. Twelve individuals (ages 9 - 53) had a subfoveal island of outer layers (width 1996.8 + 1360.1 µm), while 4 had discontinuous remnants of outer layers centrally. Perifoveal outer layers were found in 2 patients only (21 and 53 years old). Central outer layers narrowed in 23 eyes (width 1031.4 + 1057.9 µm) and were lost in 5 eyes of three patients (9 - 11 years follow up). Seven eyes were found to have cystic spaces in the ONL, six in both ONL and internal nuclear layer (INL), and 18 in the INL only (total 31 eyes; 60%). Only three eyes resolved during follow up (8 and 10 years of follow up), while three more patients developed cysts both in the ONL and INL during subsequent visits over 8 - 11 years’ time.

Six MD patients had mainly cystic changes and were therefore first thought to have X-linked retinoschisis. All 23 patients had volume scans. Five eyes went from group I to II over follow up (8-12 years). Eleven individuals (7 - 48 years old) had foveal sparing and therefore measurable central outer layers, with a mean width of 1398.1 + 1277.5 µm. Perifoveal outer layers were found in 13 patients (5 – 51 years old), where degeneration started in the macular centre. Central outer layers became discontinuous in two patients (4 and 7 years of follow up) and narrowed in six (width 747.3 + 299.6 µm). Thirteen eyes had cystic spaces both in the ONL and the INL, and eight eyes had cysts in the INL only (total 21 eyes; 44%). Seven eyes resolved during follow up (3 - 11 years of follow up).

Electrophysiology. In 4 out of 5 patients with rod-cone dystrophy, there was evidence that the DA10 electroretinogram (ERG) was dominated by a dark-adapted cone system contribution; in addition to a-wave reduction, the waveform was electronegative in 4 out of 5 and the DA red flash ERG dominated by the cone-mediated x-wave in 3 of 3 cases. One patient showed a relatively mild selective loss of rod photoreceptor function (Figure 5, patient 21). Seven patients had a macular dystrophy including 6 with normal ERGs and one with mild reduction in the LA 30z ERG (Figure 5, patient 28), but likely due to eye closure noted during testing.

Pattern ERG P50 was undetectable in 13 of 27 subjects and was subnormal in 13 others, consistent with macular dysfunction of variable severity (Figure 5b). Greatest preservation of the PERG P50 component was seen in two individuals with RP, with ERGs suggestive of a rod-cone pattern of dysfunction, including 1 with a normal PERG (Figure 5a, patient 12).

Nine of the youngest children (aged 2-8 years) were tested using lower eyelid skin electrodes according to an abbreviated ERG protocol. The photopic and scotopic ERGs were undetectable in 4 cases and were detectable but with evidence of generalised retinal dysfunction in 3 cases, all showing similar severity of rod and cone system involvement. Six of 6 of the above cases had undetectable large field pattern ERGs, in keeping with severe macular involvement. In the other cases there was evidence of mild generalised cone system dysfunction (cone dystrophy) with a detectable but subnormal pattern ERG and one had normal ERGs but a subnormal pattern ERG P50 component to a standard stimulus field, consistent with dysfunction confined to the macula.

Four patients underwent repeat ERG testing over periods of 11, 7, 5 and 1 years (supplementary figure 4). A 6-year-old child with LCA and a cone-rod pattern of dysfunction showed significant worsening of DA and LA ERGs over 11 years; a residual pattern ERG P50 component was detectable at baseline, but was undetectable 5 years later. A 20-year-old patient with MD presented with a similar degree of rod and cone system dysfunction and undetectable PERGs showed mild worsening of DA ERGs over 7 years. Two patients with MD had normal ERGs at baseline showed a high degree of ERG stability at follow-up, including a 12-year-old patient monitored over 5 years and 43-year-old woman re-tested after 1 year; baseline pattern ERG P50 was undetectable in the 12-year-old and was subnormal in the older patient, consistent with macular dystrophy in both.

1. Vincent A, Robson AG, Neveu MM, et al. A phenotype-genotype correlation study of X-linked retinoschisis. *Ophthalmology*. 2013;120(7):1454-1464. doi:10.1016/j.ophtha.2012.12.008

2. Ajamil-Rodanes S, Testi I, Luis J, Robson AG, Westcott M, Pavesio C. Evaluation of fluocinolone acetonide 0.19 mg intravitreal implant in the management of birdshot retinochoroiditis. *Br J Ophthalmol*. Published online November 2020. doi:10.1136/bjophthalmol-2020-317372
