# Supplementary material for: CRB1-Associated Retinal Dystrophies: Genetics, Clinical Characteristics, and Natural History
Source: Am J Ophthalmol. 2023 Feb;246:107–21. doi: 10.1016/j.ajo.2022.09.002 (PMC10555856; doi:10.1016/j.ajo.2022.09.002)
Supplement: Supplementary file 2 [file mmc2.pdf]

Supplementary Table 1: Clinical and genetic characteristics of patients with presumed *CRB1*-associated disease. These patients were not included in the analysis due to only one *CRB1* disease-causing allele being identified.

ID	Gender	Race	Phenotype	Family history	Age of Onset	Age first visit (years)	Presenting Symptom(s)	Age at Visit (years)	OD	OS	OCT	Macular Appearance	Peripheral Retinal appearance	CRB1 allele	CRB1 protein
**I**	M		EOSRD/LCA	Y	2 mo	3	Nystagmus	10	HM	LP	Blurred layers	Blunted, granular	Diffuse pigmented bone spicule-like	c.1576C>T	p.Arg526Ter
**II**	M	White British	EOSRD/LCA	n	2 mo	7	Nystagmus, decreased vision	11	CF	CF	Atrophic	Atrophic	Mottling	c.3655C>G	p.Gln1219Glu
**III**	F	White British	EOSRD/LCA	n	4	5	Nyctalopia	26	1.8	1.8		RPE mottling	Diffuse pigment	c.3307G>A	p.Gly1103Arg
**IV**	M		RP	n	7	35	Nyctalopia	44	HM	HM	Thickened, blurred layers	Atrophy, annular pigment	Dense bone spicule-like and nummular pigmentation, vessel thinning	c.584G>T	p.Cys195Phe
**V**	M		RP	n	18	52	Nyctalopia	40	0.5	HM	Thinned, loss of outer layers	Normal	Few bone spicule-like and nummular pigmentation, vessel thinning	c.2533_2539del	p.Gly845SerfsTer9
**VI**	M	White British	RP	n	20	26	Nyctalopia	33	0.2	0.2	Oedema	Normal	Dense bone spicule-like	c.498_506del	p.Ile167_Gly169del
**VII**	M		Macular dystrophy	n	5	5	Decreased vision	29	0.4	0.4	Loss of outer layers subfoveally	Mildly atrophic	Normal	c.3988del	p.Glu1330SerfsTer11

EOSRD/LCA: Early Onset Severe Retinal Dystrophy/ Leber Congenital Amaurosis; RP: Retinitis Pigmentosa; OD: right eye; OS: left eye; OCT: Optical Coherence Tomography; CF: count fingers; HM: hand movements; LP: light perception; F: female; M: male.
