# Supplementary material for: CRB1-Associated Retinal Dystrophies: Genetics, Clinical Characteristics, and Natural History
Source: Am J Ophthalmol. 2023 Feb;246:107–21. doi: 10.1016/j.ajo.2022.09.002 (PMC10555856; doi:10.1016/j.ajo.2022.09.002)
Supplement: Supplementary file 3 [file mmc3.pdf]

Supplementary Table 2: Quantitative macular Optical Coherence Tomography assessments (mean ± SD) at baseline and latest visits.

	Number of pts	FU time (years)	Age (years)	CMT (µm)	CMV (mm^3)	IRT (µm)	IRV (mm^3)	ORT (µm)	ORV (mm^3)	TMV (mm^3)
**EOSRD/LCA - baseline**	15	-	22 ± 15	250.4 ± 90.4	0.21 ± 0.05	372.1 ± 41.9	0.59 ± 0.06	387.2 ± 39.1	2.05 ± 0.22	10.8 ± 1.27
**EOSRD/LCA - follow up**	11	7 ± 3	28 ± 6	214.8 ± 54.2	0.17 ± 0.04	349 ± 32.48	0.55 ± 0.05	374.5 ± 55.3	1.98 ± 0.29	10.22 ± 1.21
**RP - baseline**	8	-	30.5 ± 23.5	161.6 ± 72	0.16 ± 0.05	312.5 ± 53.2	0.52 ± 0.05	360.1 ± 64.1	1.89 ± 0.12	9.85 ± 0.52
**RP - follow up**	7	7.5 ± 1.5	33 ± 10	196.3 ± 88	0.17 ± 0.06	331.1 ± 29.7	0.51 ± 0.05	365.3 ± 43.4	1.95 ± 0.15	10.2 ± 0.9
**MD - baseline**	10	-	26 ± 17	190.3 ± 68.8	0.15 ± 0.05	304.3 ± 40.2	0.47 ± 0.06	289.2 ± 43.7	1.51 ± 0.18	8.07 ± 1.06
**MD - follow up**	10	7 ± 3	31 ± 17.5	216.5 ± 73.7	0.17 ± 0.06	310.7 ± 40.8	0.48 ± 0.06	292.1 ± 38.6	1.54 ± 0.21	8.15 ± 0.97

EOSRD/LCA: Early Onset Severe Retinal Dystrophy/Leber Congenital Amaurosis; RP: Retinitis Pigmentosa; MD: Macular Dystrophy; SD: Standard Deviation; CMT: central macular thickness; CMV: central macular volume; IRT: inner ring thickness; IRV: inner ring volume; ORT: outer ring thickness; ORV: outer ring volume; TMV: total macular volume; pts: patients.
