# Supplementary material for: CRB1-Associated Retinal Dystrophies: Genetics, Clinical Characteristics, and Natural History
Source: Am J Ophthalmol. 2023 Feb;246:107–21. doi: 10.1016/j.ajo.2022.09.002 (PMC10555856; doi:10.1016/j.ajo.2022.09.002)
Supplement: Supplementary file 4 [file mmc4.pdf]

NM	Gene	Nucleotide change	Nucleotide change	Amino acid change	Position	Coding impact	Location	dbSNP ID	Verdict	ACMG Classification – Idntified classification rules: Factor 1	Factor 2	Factor 3	Factor 4	Factor 5	Factor 6	Factor 7	Factor 8	Factor 9	GnomAD allele frequency – Allele frequency (exome): East Asian	South Asian	African	European (Non-Finnish)	Latino	Total	Allele frequency (genome): East Asian	South Asian	African	European (Non-Finnish)	Total	Coverage in gnomAD Exomes samples: Mean coverage	Median coverage	% of samples over 20x coverage	MutationTaster: Prediction	Accuruacy	Converted rankscore
NM_201253.3	CRB1	c.253T>C	NM_201253.3:c.253T>C	p.Cys85Arg	197297734	missense	exon 2 of 12 position 183 of 582	rs1195391388	Likely Pathogenic	PM2	PM5	PP3	PP4						NA	NA	NA	NA	NA	NA	NA	NA	NA	NA	NA	76.2	83	99.97%	Disease causing	1	0.81
NM_201253.3	CRB1	c.254G>A	NM_201253.3:c.254G>A	p.Cys85Tyr	197297735	missense	exon 2 of 12 position 184 of 582	rs1553249226	Likely Pathogenic*	PM2	PP3	PP4	PP5						NA	NA	NA	NA	NA	NA	NA	NA	NA	NA	NA	75.9	83	99.97%	Disease causing	1	0.81
NM_201253.3	CRB1	c.481G>A	NM_201253.3:c.481G>A	p.Ala161Thr	197297962	missense	exon 2 of 12 position 411 of 582	rs1064797126	Likely Pathogenic	PM2	PM5	PP3	PP4	PP5					NA	NA	NA	NA	NA	NA	NA	NA	NA	NA	NA	59.5	55	96.59%	Disease causing	1	0.81
NM_201253.3	CRB1	c.482C>T	NM_201253.3:c.482C>T	p.Ala161Val	197297963	missense	exon 2 of 12 position 412 of 582	rs62635651	Likely Pathogenic*	PM2	PP3	PP4	PP5						NA	NA	NA	NA	NA	NA	NA	NA	NA	NA	NA	59.4	55	96.47%	Disease causing	1	0.81
NM_201253.3	CRB1	c.498 506delAATTGATG	NM_201253.3:c.498_506delAATT	p.Ile167_Gly169del	197297974	inframe	exon 2 of 12 position 428-436 of	rs398124615	Likely Pathogenic	PM2	PM4	PM3	PP4	PP5					NA	NA	0.000247	0.000968	0.000724	0.000625	NA	NA	0.00034	0.000778	0.000541	63.2	63	97.70%	NA	NA	NA
NM_201253.3	CRB1	c.584G>T	NM_201253.3:c.584G>T	p.Cys195Phe	197298065	missense	exon 2 of 12 position 514 of 582	rs764256655	Likely Pathogenic	PM2	PM3	PP4	PP5						NA	NA	0.0000615	0.0000176	NA	0.0000119	NA	NA	NA	0.0000648	0.0000318	79.3	100	99.84%	Disease causing	1	0.81
NM_201253.3	CRB1	c.2956_2957insGA	NM_201253.3:c.2956_2957insG	p.Asn986ArgfsTer3	197313476	frameshift	exon 9 of 12 before position 115	NA	Pathogenic	PVS1	PM2	PP4							NA	NA	NA	NA	NA	NA	NA	NA	NA	NA	NA	83.6	89	99.73%	NA	NA	NA
NM_201253.3	CRB1	c.717_718insG	NM_201253.3:c.717_718insG	p.Gln240AlafsTer21	197313476	frameshift	exon 3 of 12 before position 66 o	NA	Pathogenic	PVS1	PM2	PM3	PP4						NA	NA	NA	NA	NA	NA	NA	NA	NA	NA	NA	97.5	100	99.87%	NA	NA	NA
NM_201253.3	CRB1	c.750T>G	NM_201253.3:c.750T>G	p.Cys250Trp	197313508	missense	exon 3 of 12 position 98 of 196 (	rs62635652	Likely Pathogenic	PM2	PP2	PP3	PP4	PP5					NA	0.0000327	NA	NA	NA	0.000004	NA	NA	NA	NA	NA	94.1	100	99.87%	Disease causing	1	0.81
NM_201253.3	CRB1	c.988+1G>T	NM_201253.3:c.988+1G>T	NA	197316610	splicing	intron 4 of 11 position 1 of 9351 (	NA	Pathogenic	PVS1	PM2	PP4							NA	NA	NA	NA	NA	NA	NA	NA	NA	NA	NA	63.4	62	99.94%	Disease causing	1	0.81
NM_201253.3	CRB1	c.988+6T>G	NM_201253.3:c.988+6T>G	NA	197316615	splicing	intron 4 of 11 position 6 of 9351 (	NA	Unertain significance	PM2	PM3	PP4	BP4						NA	NA	NA	NA	NA	NA	NA	NA	NA	NA	NA	59.4	58	99.88%	NA	NA	NA
NM_201253.3	CRB1	c.1006T>C	NM_201253.3:c.1006T>C	p.Cys336Arg	197325978	missense	exon 5 of 12 position 18 of 183 (	rs878853370	Likely Pathogenic	PM2	PP1	PP3	PP4	PP5					NA	NA	NA	NA	NA	NA	NA	NA	NA	NA	NA	79.8	100	99.85%	Disease causing	1	0.81
NM_201253.3	CRB1	c.1183G>T	NM_201253.3:c.1183G>T	p.Glu395Ter	197390141	nonsense	exon 6 of 12 position 12 of 957 (	rs369775002	Pathogenic	PVS1	PM2	PM3	PP4	PP5					NA	NA	NA	NA	NA	NA	NA	NA	0.00023	NA	0.0000637	87.9	100	99.84%	Disease causing	1	0.81
NM_201253.3	CRB1	c.1312T>C	NM_201253.3:c.1312T>C	p.Cys438Arg	197390270	missense	exon 6 of 12 position 141 of 957	NA	Likely Pathogenic	PM2	PM3	PM5	PP3	PP4					NA	NA	NA	NA	NA	NA	NA	NA	NA	NA	NA	96.8	100	100%	Disease causing	1	0.81
NM_201253.3	CRB1	c.1429G>A	NM_201253.3:c.1429G>A	p.Gly477Arg	197390387	missense	exon 6 of 12 position 258 of 957	rs866822473	Likely Pathogenic*	PM2	PP3	PP4	PP5						NA	NA	NA	NA	NA	NA	NA	NA	NA	NA	NA	82	89	99.99%	Disease causing	1	0.81
NM_201253.3	CRB1	c.1431delG	NM_201253.3:c.1431delG	p.Ser478ProfsTer24	197390389	frameshift	exon 6 of 12 position 260 of 957	NA	Pathogenic	PVS1	PM2	PP3	PP4	PP5					NA	NA	NA	NA	NA	NA	NA	NA	NA	NA	NA	82	89	99.99%	NA	NA	NA
NM_201253.3	CRB1	c.1459T>C	NM_201253.3:c.1459T>C	p.Ser487Pro	197390417	missense	exon 6 of 12 position 288 of 957	NA	Likely Pathogenic*	PM2	PP3	PP4	PP5						NA	NA	NA	NA	NA	NA	NA	NA	NA	NA	NA	81.8	88	99.96%	Disease causing	1	0.81
NM_201253.3	CRB1	c.1576C>T	NM_201253.3:c.1576C>T	p.Arg526Ter	197390534	nonsense	exon 6 of 12 position 405 of 957	rs114342808	Pathogenic	PVS1	PM2	PP4	PP5						0.000163	NA	NA	0.0000352	NA	0.0000318	NA	NA	NA	0.0000648	0.0000318	93.9	100	100%	Disease causing	1	0.81
NM_201253.3	CRB1	c.1612_1613insCTTA	NM_201253.3:c.1612_1613insCT	p.Leu538ProfsTer14	rs87885336	frameshift	exon 6 of 12 before position 442	NA	Pathogenic	PVS1	PM2	PP4	PP5						NA	NA	NA	NA	NA	NA	NA	NA	NA	NA	NA	97.2	100	100%	NA	NA	NA
NM_201253.3	CRB1	c.1667T>C	NM_201253.3:c.1667T>C	p.Leu556Pro	197390625	missense	exon 6 of 12 position 496 of 957	rs1571524269	Likely Pathogenic	PM1	PM2	PP3	PP4	PP5					NA	NA	NA	NA	NA	NA	NA	NA	NA	NA	NA	98.6	100	100%	Disease causing	1	0.81
NM_201253.3	CRB1	c.1673T>G	NM_201253.3:c.1673T>G	p.Ile558Ser	197390631	missense	exon 6 of 12 position 502 of 957	NA	Likely Pathogenic	PM2	PM3	PP3	PP4						NA	NA	NA	NA	NA	NA	NA	NA	NA	NA	NA	NA	NA	NA	Disease causing	0.9998	0.81
NM_201253.3	CRB1	c.1696G>T	NM_201253.3:c.1696G>T	p.Glu566Ter	197390654	nonsense	exon 6 of 12 position 525 of 957	NA	Pathogenic	PVS1	PM2	PP4	PP5						NA	NA	NA	NA	NA	NA	NA	NA	NA	NA	NA	98.7	100	99.99%	Disease causing	1	0.81
NM_201253.3	CRB1	c.1712A>C	NM_201253.3:c.1712A>C	p.Glu571Ala	197390670	missense	exon 6 of 12 position 541 of 957	rs773233587	Likely Pathogenic	PM1	PM2	PM3	PP3	PP4	PP5				NA	NA	NA	0.00000881	NA	0.00000398	NA	NA	NA	NA	NA	98.5	100	99.98%	Disease causing	1	0.81
NM_201253.3	CRB1	c.1831T>C	NM_201253.3:c.1831T>C	p.Ser611Pro	197390789	missense	exon 6 of 12 position 660 of 957	rs769909288	Likely Pathogenic*	PM2	PP3	PP4	PP5						0.000381	NA	NA	NA	NA	0.0000279	0.00128	NA	NA	NA	0.0000637	98.3	100	99.95%	Disease causing	1	0.81
NM_201253.3	CRB1	c.1844G>T	NM_201253.3:c.1844G>T	p.Gly615Val	197390802	missense	exon 6 of 12 position 673 of 957	rs768905244	Likely Pathogenic	PS1	PM2	PP3	PP4	PP5					NA	NA	NA	NA	NA	NA	NA	NA	NA	NA	NA	98.6	100	99.96%	Disease causing	1	0.81
NM_201253.3	CRB1	c.2017A>G	NM_201253.3:c.2017A>G	p.Lys673Glu	197390975	missense	exon 6 of 12 position 846 of 957	rs1553260517	Likely Pathogenic*	PM2	PP3	PP4	PP5						NA	NA	NA	NA	NA	NA	NA	NA	NA	NA	NA	96.4	100	99.98%	Disease causing	0.9993	0.467
NM_201253.3	CRB1	c.2025G>T	NM_201253.3:c.2025G>T	p.Trp675Cys	197390983	missense	exon 6 of 12 position 854 of 957	NA	Likely Pathogenic*	PM2	PP3	PP4	PP5						NA	NA	NA	NA	NA	NA	NA	NA	NA	NA	NA	94.9	100	99.98%	Disease causing	1	0.81
NM_201253.3	CRB1	c.2043T>A	NM_201253.3:c.2043T>A	p.Cys681Ter	197391001	nonsense	exon 6 of 12 position 872 of 957	NA	Pathogenic	PVS1	PM2	PM3	PP4	PP5					NA	NA	NA	NA	NA	NA	NA	NA	NA	NA	NA	90.1	100	99.95%	Disease causing	1	0.81
NM_201253.3	CRB1	c.2129A>T	NM_201253.3:c.2129A>T	p.Glu710Val	197396584	missense/splic	exon 7 of 12 position 1 of 548 (sp	rs145282040	Pathogenic	PVS1	PM2	PP1	PP4	PP5					NA	NA	0.0000615	0.0000177	NA	0.0000159	NA	NA	NA	0.000129	0.0000159	57.8	57	99.47%	Disease causing	1	0.81
NM_201253.3	CRB1	c.2220dupC	NM_201253.3:c.2220dupC	p.Met741HisfsTer49	197396675	frameshift	exon 7 of 12 before position 93 o	rs1553261468	Pathogenic	PVS1	PM2	PM3	PP4	PP5					NA	NA	NA	NA	NA	NA	NA	NA	NA	NA	NA	58.5	51	98.81%	NA	NA	NA
NM_201253.3	CRB1	c.2222T>G	NM_201253.3:c.2222T>G	p.Met741Arg	197396677	missense	exon 7 of 12 position 94 of 548 (	NA	Likely Pathogenic	PM2	PM5	PP3	PP4						NA	NA	NA	NA	NA	NA	NA	NA	NA	NA	NA	58.8	52	98.92%	Disease causing	1	0.81
NM_201253.3	CRB1	c.2230C>T	NM_201253.3:c.2230C>T	p.Arg744Ter	197396685	nonsense	exon 7 of 12 position 102 of 548	rs150412614	Pathogenic	PVS1	PM2	PM3	PP4	PP5					NA	NA	NA	0.0000264	NA	0.0000119	NA	NA	NA	NA	NA	58.5	51	99.04%	Disease causing	1	0.81
NM_201253.3	CRB1	c.2234C>T	NM_201253.3:c.2234C>T	p.Thr745Met	197396689	missense	Unertain significance	rs28939720	Likely Pathogenic	PM2	PM3	PM5	PP3	PP4	PP5				0.0000544	0.0000654	0.000123	0.000106	NA	0.0000678	NA	NA	NA	0.0000649	0.0000319	58.6	51	99.04%	Disease causing	1	0.81
NM_201253.3	CRB1	c.2272A>T	NM_201253.3:c.2272A>T	p.Ser758Cys	197396727	missense	exon 7 of 12 position 144 of 548	rs201700675	Unertain significance	PM2	PP3	PP4							NA	NA	NA	0.0000353	NA	0.0000199	NA	NA	NA	NA	NA	59.5	51	99.24%	Disease causing	1	0.81
NM_201253.3	CRB1	c.2290C>T	NM_201253.3:c.2290C>T	p.Arg764Cys	197396745	missense	exon 7 of 12 position 162 of 548	rs62635654	Likely Pathogenic	PM2	PM3	PM5	PP1	PP4	PP5				0.0000544	0.0000654	NA	0.000106	0.000087	0.0000757	NA	NA	NA	0.000194	0.0000955	62.9	57	98.97%	NA	NA	NA
NM_201253.3	CRB1	c.2291G>A	NM_201253.3:c.2291G>A	p.Arg764His	197396746	missense	exon 7 of 12 position 163 of 548	rs375040930	Likely Pathogenic	PM2	PM5	PP4	PP5						NA	0.0000327	0.0000616	NA	NA	0.0000119	NA	NA	NA	0.0000648	0.0000637	63.3	58	98.99%	Polymorphism	1	0.08975
NM_201253.3	CRB1	c.2308G>C	NM_201253.3:c.2308G>C	p.Gly770Arg	197396763	missense	exon 7 of 12 position 180 of 548	rs767648174	Likely Pathogenic	PM2	PM5	PP3	PP4	PP5					NA	NA	NA	0.00000883	NA	0.00000399	NA	NA	NA	NA	NA	66.5	69	98.59%	Disease causing	1	0.81
NM_201253.3	CRB1	c.2308G>T	NM_201253.3:c.2308G>T	p.Gly770Cys	197396763	missense	exon 7 of 12 position 180 of 548	NA	Likely Pathogenic	PM1	PM2	PM5	PP3	PP4					NA	NA	NA	NA	NA	NA	NA	NA	NA	NA	NA	66.5	69	98.59%	Disease causing	1	0.81
NM_201253.3	CRB1	c.2358del	NM_201253.3:c.2358del	p.Phe786LeufsTer10	197396813	frameshift	exon 7 of 12 position 230 of 548	NA	Pathogenic	PVS1	PM2	PP4							NA	NA	NA	NA	NA	NA	NA	NA	NA	NA	NA	67.8	79	97.04%	Disease causing	1	0.81
NM_201253.3	CRB1	c.2401A>T	NM_201253.3:c.2401A>T	p.Lys801Ter	197396856	nonsense	exon 7 of 12 position 273 of 548	rs137853137	Pathogenic	PVS1	PM2	PM3	PP4	PP5					NA	NA	0.0000618	0.0000883	NA	0.0000519	NA	NA	NA	0.000194	0.0000955	65.9	66	98.42%	Disease causing	1	0.81
NM_201253.3	CRB1	c.2491T>C	NM_201253.3:c.2491T>C	p.Tyr831His	197396946	missense	exon 7 of 12 position 363 of 548	rs1394180573	Likely Pathogenic	PM2	PM3	PP2	PP4						NA	NA	NA	NA	NA	NA	NA	NA	NA	NA	NA	81.2	100	99.68%	Disease causing	1	0.81
NM_201253.3	CRB1	c.2506C>A	NM_201253.3:c.2506C>A	p.Pro836Thr	197396961	missense	exon 7 of 12 position 378 of 548	rs116471343	Unertain significance**	PM2	PP1	PP3	PP4	PP5					NA	NA	0.00277	NA	0.000203	0.000211	NA	NA	0.00287	NA	0.000828	81.9	100	99.71%	Disease causing	1	0.81
NM_201253.3	CRB1	c.2533_2539del	NM_201253.3:c.2533_2539del	p.Gly845SerfsTer9	197396986	frameshift	exon 7 of 12 position 405-411 of	rs745348555	Pathogenic	PVS1	PM2	PP4	PP5						NA	NA	NA	0.00000881	NA	0.00000398	NA	NA	NA	NA	NA	82.1	100	99.76%	Disease causing	1	0.81
NM_201253.3	CRB1	c.2536G>A	NM_201253.3:c.2536G>A	p.Gly846Arg	197396991	missense	exon 7 of 12 position 408 of 548	rs539189291	Pathogenic	PS1	PM2	PM5	PP3	PP4	PP5				NA	0.000098	NA	NA	NA	0.0000119	NA	NA	NA	NA	NA	81.2	95	99.75%	Disease causing	1	0.81
NM_201253.3	CRB1	c.2548G>A	NM_201253.3:c.2548G>A	p.Gly850Ser	197397003	missense	exon 7 of 12 position 420 of 548	rs776591659	Likely Pathogenic	PM2	PM3	PM5	PP3	PP4	PP5				NA	NA	0.0000615	0.0000352	0.0000289	0.0000239	NA	NA	NA	0.0000648	0.0000319	81.6	100	99.73%	Disease causing	1	0.81
NM_201253.3	CRB1	c.2555T>C	NM_201253.3:c.2555T>C	p.Ile852Thr	197397010	missense	exon 7 of 12 position 427 of 548	rs62636271	Likely Pathogenic	PM2	PM3	PP1	PP3	PP4	PP5				NA	NA	NA	0.00000881	NA	0.00000398	NA	NA	NA	NA	NA	80.9	98	99.73%	Disease causing	1	0.81
NM_201253.3	CRB1	c.2639A>G	NM_201253.3:c.2639A>G	p.Asn880Ser	197397094	missense	exon 7 of 12 position 511 of 548	rs910489135	Likely Pathogenic	PM2	PM5	PP1	PP3	PP4	PP5				NA	NA	NA	NA	NA	NA	NA	NA	NA	NA	NA	74.3	76	99.58%	Disease causing	1	0.81
NM_201253.3	CRB1	c.2673C>A	NM_201253.3:c.2673C>A	p.Cys891Ter	197397128	nonsense	exon 7 of 12 position 545 of 548	rs759662695	Pathogenic	PVS1	PM2	PM3	PP5						NA	NA	NA	NA	NA	NA	NA	NA	NA	NA	NA	67.9	66	99.15%	Disease causing	1	0.81
NM_201253.3	CRB1	c.2676delG	NM_201253.3:c.2676delG	p.Lys892AsnfsTer96	197397131	frameshift	intron 7 of 11 position 1 of 1447 (	NA	Pathogenic	PVS1	PM2	PP4							NA	NA	NA	NA	NA	NA	NA	NA	NA	NA	NA	67.1	65	99.04%	NA	NA	NA
NM_201253.3	CRB1	c.2676G>C	NM_201253.3:c.2676G>C	p.Lys892Asn	197397131	missense/splic	exon 7 of 12 position 548 of 548	NA	Likely Pathogenic	PVS1	PM2	PP1	PP2	PP3	PP4				NA	NA	NA	NA	NA	NA	NA	NA	NA	NA	NA	67.1	65	99.04%	Disease causing	1	0.81
NM_201253.3	CRB1	c.2680A>C	NM_201253.3:c.2680A>C	p.Asn894His	197398582	missense	exon 8 of 12 position 4 of 166 (s	NA	Unertain significance	PM2	PP3	PP4							NA	NA	NA	NA	NA	NA	NA	NA	NA	NA	NA	97.6	100	99.98%	Disease causing	0.8259	0.81
NM_201253.3	CRB1	c.2688T>A	NM_201253.3:c.2688T>A	p.Cys896Ter	197398590	nonsense	exon 8 of 12 position 12 of 166 (	rs62636273	Pathogenic	PVS1	PM2	PM3	PP4	PP5					NA	NA	NA	0.0000528	0.0000578	0.0000318	NA	NA	NA	NA	0.0000318	97.3	100	99.99%	Disease causing	1	0.81
NM_201253.3	CRB1	c.2839G>A	NM_201253.3:c.2839G>A	p.Glu947Lys	197398741	missense	exon 8 of 12 position 163 of 166	rs574162883	Likely Pathogenic	PM1	PM2	PP3	PP4	PP5					NA	0.000653	NA	0.00000881	NA	0.0000836	NA	NA	NA	NA	NA	60.1	58	99.44%	Disease causing	1	0.81
NM_201253.3	CRB1	c.2843-1G>A	NM_201253.3:c.2843-1G>A	NA	197403835	splicing	intron 8 of 11 position 5091 of 50	rs878853368	Pathogenic	PVS1	PM2	PM3	PP4						NA	NA	NA	NA	NA	NA	NA	NA	NA	NA	NA	66.9	65	99.12%	Disease causing	1	0.81
NM_201253.3	CRB1	c.2843G>A	NM_201253.3:c.2843G>A	p.Cys948Tyr	197403836	missense/splic	exon 9 of 12 position 1 of 907 (s	rs62645748	Pathogenic	PVS1	PM2	PM3	PP4	PP5					0.0000544	NA	0.0000637	0.000407	0.000116	0.000212	NA	NA	NA	0.000259	0.000127	67.7	66	99.22%	Disease causing	1	0.81
NM_201253.3	CRB1	c.3008T>C	NM_201253.3:c.3008T>C	p.Ile1003Thr	197404001	missense	exon 9 of 12 position 166 of 907	rs1409740542	Likely Pathogenic*	PM2	PP3	PP4	PP5						NA	NA	NA	0.00000883	NA	0.00000399	NA	NA	NA	NA	NA	81.8	87	99.67%	Disease causing	1	0.81
NM_201253.3	CRB1	c.3014A>T	NM_201253.3:c.3014A>T	p.Asp1005Val	197404007	missense	exon 9 of 12 position 172 of 907	rs369184026	Likely Pathogenic*	PM2	PP3	PP4	PP5						NA	NA	NA	0.0000529	NA	0.0000239	NA	NA	NA	NA	NA	81.6	86	99.68%	Disease causing	1	0.81
NM_201253.3	CRB1	c.3035T>C	NM_201253.3:c.3035T>C	p.Leu1012Ser	197404028	missense	exon 9 of 12 position 193 of 907	NA	Likely Pathogenic	PM2	PP2	PP3	PP4	PP5					NA	NA	NA	NA	NA	NA	NA	NA	NA	NA	NA	82.4	88	99.70%	Disease causing	1	0.81
NM_201253.3	CRB1	c.3037C>T	NM_201253.3:c.3037C>T	p.Gln1013Ter	197404030	nonsense	exon 9 of 12 position 195 of 907	rs143511261	Pathogenic	PVS1	PM2	PP4	PP5						NA	NA	NA	0.00000883	NA	0.00000399	NA	NA	NA	NA	NA	82.4	88	99.69%	Disease causing	1	0.81
NM_201253.3	CRB1	c.3074G>A	NM_201253.3:c.3074G>A	p.Ser1025Asn	197404067	missense	exon 9 of 12 position 232 of 907	NA	Likely Pathogenic	PM2	PM5	PP4	PP5						NA	NA	NA	NA	NA	NA	NA	NA	NA	NA	NA	91.9	100	99.79%	Disease causing	1	0.81
NM_201253.3	CRB1	c.3307G>A	NM_201253.3:c.3307G>A	p.Gly1103Arg	197404300	missense	exon 9 of 12 position 465 of 907	rs62636275	Likely Pathogenic	PM2	PM3	PM5	PP3	PP4	PP5				NA	0.0000327	NA	0.0000532	NA	0.0000279	NA	NA	NA	NA	NA	91.5	100	99.42%	Disease causing	0.9999	0.5876
NM_201253.3	CRB1	c.3320T>C	NM_201253.3:c.3320T>C	p.Leu1107Pro	197404313	missense	exon 9 of 12 position 478 of 907	rs62636276	Likely Pathogenic	PM2	PM5	PP3	PP4	PP5					NA	NA	NA	0.00000886	NA	0.00000399	NA	NA	NA	NA	NA	91.7	100	99.44%	Disease causing	1	0.81
NM_201253.3	CRB1	c.3457del	NM_201253.3:c.3457del	p.Asn1153ThrfsTer57	197404450	frameshift	exon 9 of 12 position 616 of 907	NA	Pathogenic	PVS1	PM2	PP4							NA	NA	NA	NA	NA	NA	NA	NA	NA	NA	NA	72.5	70	99.76%	NA	NA	NA
NM_201253.3	CRB1	c.3520T>G	NM_201253.3:c.3520T>G	p.Cys1174Gly	197404513	missense	exon 9 of 12 position 678 of 907	rs917768074	Likely Pathogenic	PM1	PM2	PP3	PP4	PP5					NA	NA	NA	NA	NA	NA	NA	NA	NA	NA	NA	55.1	48	92.00%	Disease causing	1	0.81
NM_201253.3	CRB1	c.3542dup	NM_201253.3:c.3542dup	p.Cys1181TrpfsTer13	197404535	frameshift	exon 9 of 12 before position 701	rs1553263218	Pathogenic	PVS1	PM2	PP4	PP5						NA	NA	NA	NA	NA	NA	NA	NA	NA	NA	NA	53.1	47	86.65%	NA	NA	NA
NM_201253.3	CRB1	c.3625G>A	NM_201253.3:c.3625G>A	p.Val1209Met	197404618	missense	exon 9 of 12 position 783 of 907	rs201349525	Unertain significance	PM2	PP3	PP4							NA	NA	0.0000586	0.0000361	NA	0.0000287	NA	NA	NA	NA	NA	48.7	43	86.85%	Disease causing	1	0.81
NM_201253.3	CRB1	c.3655C>G	NM_201253.3:c.3655T>G	p.Gln1219Glu	197404648	missense	exon 9 of 12 position 813 of 907	NA	Unertain significance	PM2	PP4	BP4							NA	NA	NA	NA	NA	NA	NA	NA	NA	NA	NA	46.7	43	93.16%	Polymorphism	0.9718	0.3813
NM_201253.3	CRB1	c.3655C>T	NM_201253.3:c.3655C>T	p.Gln1219Ter	197404648	nonsense	exon 9 of 12 position 813 of 907	NA	Pathogenic	PVS1	PM2	PP4							NA	NA	NA	NA	NA	NA	NA	NA	NA	NA	NA	46.7	43	93.16%	Disease causing	1	0.81
NM_201253.3	CRB1	c.3676G>T	NM_201253.3:c.3676G>T	p.Gly1226Ter	197404669	nonsense	exon 9 of 12 position 834 of 907	rs757740068	Pathogenic	PVS1	PM2	PP4	PP5						0.000272	NA	NA	NA	NA	0.0000199	NA	NA	NA	NA	NA	45.1	40	96.71%	Disease causing	1	0.81
NM_201253.3	CRB1	c.3879-1203C>G	NM_201253.3:c.3879-1203C>G	NA	197410093	intron	intron 10 of 11 position 2288 of 3	rs999515974	Unertain significance	PM2	PM3	PP1	BP4						NA	NA	NA	0.0000649	NA	0.0000319	NA	NA	NA	NA	NA	31.6	31	95.12%	NA	NA	NA
NM_201253.3	CRB1	c.3914C>T	NM_201253.3:c.3914C>T	p.Pro1305Leu	197411331	missense	exon 11 of 12 position 36 of 127	rs1391910861	Likely Pathogenic	PM2	PM3	PP3	PP4	PP5					NA	NA	NA	NA	NA	0.000163	NA	NA	NA	NA	NA	97.4	100	99.99%	Disease causing	1	0.81
NM_201253.3	CRB1	c.3988delG	NM_201253.3:c.3988delG	p.Glu1330SerfsTer11	197411405	frameshift	exon 11 of 12 position 110 of 127	rs1057520152	Pathogenic	PVS1	PM2	PM3	PP4	PP5					NA	NA	NA	NA	NA	NA	NA	NA	NA	NA	NA	89.4	100	99.98%	NA	NA	NA
NM_201253.3	CRB1	c.4006-10A>G	NM_201253.3:c.4006-10A>G	NA	197446784	intron	intron 11 of 11 position 35362 of	rs766850702	Likely Pathogenic	PM2	PM3	PP3	PP4						NA	NA	NA	0.0000177	NA	0.00000798	NA	NA	NA	NA	NA	76.2	100	99.36%	NA	NA	NA
NM_201253.3	CRB1	c.4006-1G>A	NM_201253.3:c.4006-1G>A	NA	197446793	splicing	intron 11 of 11 position 35371 of	rs752804194	Pathogenic	PVS1	PM2	PP1	PP4						NA	NA	NA	0.00000885	NA	0.00000399	NA	NA	NA	NA	NA	77.9	100	99.59%	Disease causing	1	0.81
NM_201253.3	CRB1	c.4006-1G>T	NM_201253.3:c.4006-1G>T	NA	197446793	splicing	intron 11 of 11 position 35371 of	rs752804194	Pathogenic	PVS1	PM2	PP4	PP5						NA	NA	NA	0.00000885	NA	0.00000399	NA	NA	NA	NA	NA	77.9	100	99.59%	Disease causing	1	0.81
NM_201253.3	CRB1	c.4009 4015delGCAGAT	NM_201253.3:c.4009_4015delc.	p.Ala1337ThrfsTer2	197446797	frameshift	exon 12 of 12 position 4-10 of 79	NA	Pathogenic	PVS1	PM2	PP4							NA	NA	NA	NA	NA	NA	NA	NA	NA	NA	NA	78.2	100	99.59%	NA	NA	NA
NM_201253.3	CRB1	c.4082T>C	NM_201253.3:c.4082T>C	p.Leu1361Pro	197446870	missense	exon 12 of 12 position 77 of 792	NA	Likely Pathogenic	PM2	PM3	PP3	PP4						NA	NA	NA	NA	NA	NA	NA	NA	NA	NA	NA	83.3	100	99.79%	Disease causing	1	0.81
NM_201253.3	CRB1	c.4094C>A	NM_201253.3:c.4094C>A	p.Ala1365Asp	197446882	missense	exon 12 of 12 position 89 of 792	NA	Likely Pathogenic	PM2	PM3	PP4	PP5						NA	NA	NA	NA	NA	NA	NA	NA	NA	NA	NA	84.2	100	99.81%	Disease causing	1	0.81
NM_201253.3	CRB1	c.4142C>G	NM_201253.3:c.4142C>G	p.Pro1381Arg	197446930	missense	exon 12 of 12 position 137 of 79	NA	Likely Pathogenic	PM2	PM5	PP3	PP4	PP5					NA	NA	NA	NA	NA	NA	NA	NA	NA	NA	NA	89.5	100	99.83%	Disease causing	1	0.81
NM_201253.3	CRB1	c.4142C>T	NM_201253.3:c.4142C>T	p.Pro1381Leu	197446930	missense	exon 12 of 12 position 137 of 79	NA	Likely Pathogenic	PM2	PM5	PP3	PP4	PP5					NA	NA	NA	NA	NA	NA	NA	NA	NA	NA	NA	89.5	100	99.83%	Disease causing	1	0.81
NM_201253.3	CRB1	exon 12 deletion			NA	large deletion	deletion of exon 12	NA	Pathogenic	PVS1	PM2								NA	NA	NA	NA	NA	NA	NA	NA	NA	NA	NA	NA	NA	NA	NA	NA	NA
NM_201253.3	CRB1	c.2129-894T>A	NM_201253.3:c.2129-894G>A		NA	Intron	c.2129-894T>A	NA	Unertain significance	PM2	BP4								NA	NA	NA	NA	NA	NA	NA	NA	NA	NA	NA	NA	NA	NA	NA	NA	NA

Footnote 1.

Sequence variant nomenclature was obtained according to the guidelines of the Human Genome Variation Society (HGVS) by using Mutalyzer (https://mutalyzer.nl/). All detected variants were analysed using two general prediction programs and three functional prediction programs; Mutation Taster (http://www.mutationtaster.org/), FATHMM (http://fathmm.biocompute.org.uk/9), SIFT (https://www.sift.co.uk/), PROVEAN (http://provean.jcvi.org/index.php), and Polyphen 2 (http://genetics.bwh.harvard.edu/pph2/). Minor allele frequency for the identified variants in the general population was assessed in the Genome Aggregation Database (gnomAD) datasets(https://gnomad.broadinstitute.org/). The population data and general coverage were also provided with the gnomAD database. Evolutionary conservation scores were calculated referring to the UCSC database (https://genome.ucsc.edu/index.html). Classification of predictions by the American College of Medical Genetics and Genomics (ACMG) was also applied for all detected variants. All databases were accessed on 1 November 2021. Pathogenicity classification was assessed according to the following factors: PVS1 (null variant (nonsense, frameshift, canonical ±1 or 2 splice sites, initiation codon, single or multiexon deletion) in a gene where loss of function is a known mechanism of disease); PS2 (pathogenicity supportive 2; de novo (both maternity and paternity confirmed) in a patient with the disease and no family history); PS3 (pathogenicity supportive 3; well-established in vitro or in vivo functional studies supportive of a damaging effect on the gene or gene product); PM1 (pathogenicity moderate 1; located in a mutational hot spot and/or critical and well-established functional domain without benign variation); PM2 (pathogenicity moderate 2; absent or very rare from controls in Exome Sequencing Project, 1000 Genomes Project, or Exome Aggregation Consortium); PM5 (pathogenicity moderate 5; novel missense change at an amino acid residue where a different missense change determined to be pathogenic has been seen before); PP1 (pathogenicity supporting 1; co-segregation with disease in multiple affected family members in a gene definitively known to cause the disease); PP3 (pathogenicity supporting 3; multiple lines of computational evidence support a deleterious effect on the gene or gene product); PP5 (pathogenicity supporting 5; reputable source recently reports variant as pathogenic, but the evidence is not available from the laboratory to perform an independent evaluation). All databases were accessed on 1 November 2022.

Footnote 2.

*For the purpose of this study, an additional specification (Likely pathogenic: 1 Moderate (PM1-PM6) AND 3 Supporting (PP1-PP5)) to determine the verdict assessment results was applied to the ACMG classification. In specific, 15 variants with an asterisk were classified into likely pathogenic with this additional specification.

**Allele frequency (<0.001) provided on the gnomAD exome in the total population was applied for the assessment of PM2 (absence or very are in the general population database). One variant (c.2506C>A, (p.Pro836Thr)) with a relatively high allele frequency (>0.002) in the African population was classified into uncertain significant, although the allele frequency of the gnomAD exome total was less than 0.001.

For the purpose of this study, the presence of distinctive and/or characteristic features of CRB1-retinopathy was evaluated for the assessment of PP4: nummular pigmented deposits, admixed with small yellow-white dots, preserved para-arteriolar retinal pigment epithelium, abnormally laminated and thickened retina, and peripheral exudative retinal telangiectasia (Coats-like vasculopathy).

FATHMM			FATHMM MKL			FATHMM XF			CADD	REVEL			SIFT			Functional prediction PROVEAN			Polyphen2		Human Splice Finder 3.0	Conservation PhyloP30way		PhastCons30way		Conservation PhyloP100way		PhastCons100way						
Prediction	Score	Converted rankscor	Prediction	Score	Converted rankscor	Prediction	Score	Converted rankscor	Score	Prediction	Score	Rankscore	Prediction	Score	Converted rankscor	Prediction	Score	Converted rankscor	Prediction	Score		Mammalian	Mammalian rankscor	Mammalian	Mammalian rankscor	vertebrate	vertebrate rankscor	vertebrate	vertebrate rankscor	Report name	Year	Report name	Year	
Damaging	-12.4	0.9999	Damaging	0.9794	0.783	Damaging	0.8819	0.8095	24.1	Pathogeni	0.9309	0.984	deleteriou	0	0.9125	Damaging	-9.25	0.9829	probably_damaging	0.997		1.0859	0.5419	0.933	0.4008	3.427	0.5255	0.999	0.4266	This study				Novel
Damaging	-12.4	0.9999	Damaging	0.9744	0.7477	Damaging	0.8575	0.7748	24.3	Pathogeni	0.8799	0.9652	deleteriou	0	0.9125	Damaging	-7.25	0.9726	probably_damaging	0.997		1.12	0.5652	0.934	0.4018	4.9289	0.6316	1	0.7164	Carss KJ, Arno G, Erwood M,	2016			
Damaging	-3.38	0.9416	Damaging	0.9925	0.9344	Damaging	0.8679	0.7879	24	Pathogeni	0.7979	0.9348	Damaging	0.003	0.6824	Damaging	-3.39	0.6689	probably_damaging	0.999		1.12	0.5652	0.7739	0.3276	9.4799	0.9688	1	0.7164	This study				Novel
Damaging	-3.41	0.9431	Damaging	0.9879	0.8685	Damaging	0.8651	0.7842	24.2	Pathogeni	0.827	0.9458	Damaging	0.003	0.6824	Damaging	-3.28	0.6689	probably_damaging	0.999		0.9789	0.3609	0.554	0.2814	7.4759	0.8005	1	0.7164	den Hollander AI, ten Brink [illegible]	1999			
NA	NA	NA	NA	NA	NA	NA	NA	NA	15.23	NA	NA	NA	NA	NA	NA	NA	NA	NA	NA	NA		NA	NA	NA	NA	NA	NA	NA	NA	Khan KN, Robson A, Mahroo (	2018			
Damaging	-12.4	0.9999	Damaging	0.9657	0.6981	Damaging	0.8929	0.8304	23.9	Pathogeni	0.953	0.9917	deleteriou	0	0.9125	Damaging	-9.81	0.9868	probably_damaging	0.998		1.12	0.5652	0.9919	0.541	7.335	0.7849	1	0.7164	den Hollander AI, ten Brink JB	1999			
NA	NA	NA	NA	NA	NA	NA	NA	NA	NA	NA	NA	NA	NA	NA	NA	NA	NA	NA	NA	NA		NA	NA	NA	NA	NA	NA	NA	NA	This study				Novel
NA	NA	NA	NA	NA	NA	NA	NA	NA	28.3	NA	NA	NA	NA	NA	NA	NA	NA	NA	NA	NA		NA	NA	NA	NA	NA	NA	NA	NA	Henderson RH, Mackay DS, L	2011			
Damaging	-12.4	0.9999	Neutral	0.2726	0.2323	Damaging	0.5417	0.5577	23.1	Pathogeni	0.87	0.9615	deleteriou	0	0.9125	Damaging	-8.43	0.9835	NA	NA		-0.1509	0.1092	0.5279	0.2771	0.193	0.1692	0.023	0.1992	den Hollander AI, ten Brink JB	1999	Henderso	2011	
NA	NA	NA	Damaging	0.9939	0.9537	NA	NA	NA	34	NA	NA	NA	NA	NA	NA	NA	NA	NA	NA	NA		1.1759	0.7892	1	0.8628	9.1529	0.9385	1	0.7164	This study				Novel
NA	NA	NA	NA	NA	NA	NA	NA	NA	18.62	NA	NA	NA	NA	NA	NA	NA	NA	NA	NA	NA		NA	NA	NA	NA	NA	NA	NA	NA	This study				Novel
Damaging	-3.97	0.9624	Damaging	0.9549	0.6492	Damaging	0.8056	0.7301	25.3	Pathogeni	0.953	0.9917	deleteriou	0.01	0.9125	Damaging	-9.35	0.9917	probably_damaging	0.998		1.085	0.5411	0.9969	0.6203	6.598	0.74	1	0.716	Carss KJ, Arno G, Erwood M,	2016			
NA	NA	NA	Damaging	0.9605	0.6729	NA	NA	NA	37	NA	NA	NA	NA	NA	NA	NA	NA	NA	NA	NA		1.1759	0.7892	0.9959	0.5952	3.845	0.5559	1	0.7164	Carss KJ, Arno G, Erwood M,	2016			
Damaging	-3.19	0.9317	Damaging	0.9889	0.8829	Damaging	0.9553	0.973	27.1	Pathogeni	0.934	0.9851	deleteriou	0	0.9125	Damaging	-10.8	0.9943	NA	NA		1.1089	0.5543	0.856	0.3548	7.59	0.8192	1	0.7164	NA				
Damaging	-4.79	0.9815	Damaging	0.9934	0.9472	Damaging	0.9422	0.9464	23	Pathogeni	0.902	0.9733	NA	NA	0.7215	Damaging	-6.75	0.9269	NA	NA		1.12	0.5652	0.024	0.1464	9.487	0.9692	1	0.7164	Abu-Safieh L, Alrashed M, Ana	2013			
NA	NA	NA	NA	NA	NA	NA	NA	NA	NA	NA	NA	NA	NA	NA	NA	NA	NA	NA	NA	NA		NA	NA	NA	NA	NA	NA	NA	NA	Khan KN, Robson A, Mahroo (	2018			
NA	NA	NA	Damaging	0.9746	0.7488	Damaging	0.9291	0.9142	22.6	Pathogeni	0.851	0.9546	NA	NA	0.7215	Damaging	-4.55	0.7881	NA	NA		1.0859	0.5419	0.4199	0.2599	5.8379	0.6909	1	0.7164	Eisenberger T, Neuhaus C, Kh	2013			
NA	NA	NA	Neutral	0.4316	0.2704	Neutral	0.2317	0.3549	33	NA	NA	NA	NA	NA	NA	NA	NA	NA	NA	NA		0.071	0.1531	0.2939	0.2393	0.536	0.2284	0.005	0.1704	Seong MW, Kim SY, Yu YS, H	2008	Henderso	2011	
NA	NA	NA	NA	NA	NA	NA	NA	NA	NA	NA	NA	NA	NA	NA	NA	NA	NA	NA	NA	NA		NA	NA	NA	NA	NA	NA	NA	NA	This study				Novel
Damaging	-1.54	0.8164	Damaging	0.9951	0.9691	Damaging	0.9005	0.8461	23.7	Pathogeni	0.6809	0.8845	Damaging	0.007	0.7215	Damaging	-4.04	0.7698	probably_damaging	1		1.0859	0.5419	0.026	0.1489	7.59	0.8192	1	0.7164	This study				Novel
Tolerated	-1.02	0.763	NA	NA	NA	Neutral	0.4616	0.5113	21.9	Benign	Benign	0.7589	Damaging	0.003	0.6824	NA	NA	NA	Damaging	0.999		1.0859	0.5419	0.039	0.1619	4.6069	0.6084	0.6949	0.2858	This study				Novel
NA	NA	NA	Damaging	0.9736	0.7425	Neutral	0.09856	0.1986	33	NA	NA	NA	NA	NA	NA	NA	NA	NA	NA	NA		-0.237	0.08622	0.379	0.2534	3.1449	0.5032	1	0.7164	NA				
Tolerated	-1.14	0.7784	Damaging	0.9774	0.7676	Damaging	0.7116	0.665	25	Benign	0.5839	0.835	Tolerated	0.077	0.3498	Damaging	-4.21	0.7578	probably_damaging	0.998		1.25	0.8558	0.9929	0.5512	4.767	0.6198	1	0.7164	Carss KJ, Arno G, Erwood M,	2017			
Tolerated	-1.13	0.7772	Damaging	0.9005	0.5093	Damaging	0.7048	0.6604	23.6	Pathogeni	0.683	0.8854	Damaging	0.042	0.4164	Damaging	-2.6	0.5792	NA	NA		1.0859	0.5419	0.9969	0.6203	2.0069	0.405	0.994	0.3829	Li L, Xiao X, Li S, et al. Detect	2011			
Damaging	-4.39	0.9739	Damaging	0.9959	0.9777	Damaging	0.9299	0.9165	27.4	Pathogeni	0.873	0.9626	Damaging	0.002	0.7215	Damaging	-6.8	0.9548	probably_damaging	0.996		1.12	0.5652	0.9829	0.4875	9.5659	0.9729	1	0.7164	This study				Novel
Tolerated	-1.01	0.7617	Damaging	0.903	0.5137	Neutral	0.2735	0.3889	22.7	Benign	0.314	0.6395	deleteriou	0.02	0.5125	NA	NA	NA	probably_damaging	0.989		1.25	0.8558	0.9909	0.5322	1.25	0.8558	0.998	0.4132	Carss KJ, Arno G, Erwood M,	2016			
Tolerated	0.01	0.7521	Damaging	0.9343	0.5819	Damaging	0.7234	0.673	25.1	Benign	0.425	0.7359	deleteriou	0.01	0.7215	Damaging	-9.68	0.986	probably_damaging	0.998		1.12	0.5652	1	0.8628	3.595	0.5376	1	0.7164	Henderson RH, Mackay DS, L	2011			
NA	NA	NA	Damaging	0.9045	0.5164	Neutral	0.3262	0.4275	NA	NA	NA	NA	NA	NA	NA	NA	NA	NA	NA	NA		0.163	0.2058	0.9969	0.6203	1.4919	0.352	1	0.7164	Consugar MB, Navarro-Gomez	2015	Henderso	2011	
Tolerated	-1.28	0.7938	Damaging	0.967	0.7048	Damaging	0.6626	0.6324	23.4	Pathogeni	0.6499	0.8695	deleteriou	0	0.9125	Damaging	-6.24	0.9144	probably_damaging	0.937		1.312	0.9471	0.023	0.145	1.312	0.9471	1	0.7164	Clark GR, Crowe P, Muszynsk	2010			
NA	NA	NA	NA	NA	NA	NA	NA	NA	NA	NA	NA	NA	NA	NA	NA	NA	NA	NA	NA	NA		NA	NA	NA	NA	NA	NA	NA	NA	Carss KJ, Arno G, Erwood M,	2017			
Tolerated	-1.34	0.7994	Damaging	0.9786	0.7771	Damaging	0.7432	0.6866	24.6	Pathogeni	0.651	0.87	deleteriou	0.002	0.7215	Damaging	-4.3	0.7649	possibly_damaging	0.69		1.1089	0.5543	0.6	0.2894	5.7569	0.6842	1	0.7164	This study				Novel
NA	NA	NA	Damaging	0.9317	0.5752	Neutral	0.2815	0.395	35	NA	NA	NA	NA	NA	NA	NA	NA	NA	NA	NA		0.079	0.1564	0.2029	0.222	1.7799	0.3826	0.998	0.4132	Kuniyoshi K, Ikeo K, Sakuram	2015			
Damaging	-2.55	0.8956	Damaging	0.9791	0.7808	Damaging	0.6704	0.6375	23.1	Pathogeni	0.8069	0.9382	deleteriou	0.001	0.7849	Damaging	-4.67	0.8111	probably_damaging	0.999		1	0.3687	0.1019	0.1948	5.6659	0.6773	1	0.7164	Shanks ME, Downes SM, Cop	2013			
Tolerated	-1.19	0.7843	Damaging	0.8855	0.4852	Neutral	0.321	0.4239	17.96	Benign	0.4199	0.7322	deleteriou	0.003	0.6824	Damaging	-3.89	0.7389	possibly_damaging	0.873		1.2779	0.8601	0.465	0.2671	1.465	0.3491	0.9909	0.3726	This study				Novel
Tolerated	-1.16	0.7808	Neutral	0.09847	0.1551	Neutral	0.1892	0.3165	14.95	Benign	0.4749	0.7701	tolerated	0.059	0.3863	Damaging	-3.91	0.7304	benign	0.007		-0.3389	0.06944	0.206	0.2227	0.8019	0.2673	0.01799	0.1946	Henderson RH, Mackay DS, L	2011			
Tolerated	-1.13	0.7772	Neutral	0.08332	0.1427	Neutral	0.08579	0.1739	14.25	Benign	0.5299	0.8042	tolerated	0.16	0.2942	NA	NA	NA	possibly_damaging	0.708		1.146	0.7025	0.012	0.2368	0.2829	0.2829	0.004	0.1661	Corton M, Tatu SD, Avila-Fern	2013			
Damaging	-2.13	0.8635	Damaging	0.9942	0.9576	Damaging	0.8875	0.8198	23.8	Pathogeni	0.8769	0.9641	deleteriou	0	0.9125	Damaging	-7.15	0.9479	probably_damaging	1		1.146	0.7025	0.01	0.1189	7.5149	0.8066	1	0.7164	Eisenberger T, Neuhaus C, Kh	2014			
Damaging	-2.17	0.8662	Damaging	0.9938	0.9534	Damaging	0.9067	0.8599	24.1	Pathogeni	0.85	0.9543	deleteriou	0	0.9125	Damaging	-8.03	0.9702	probably_damaging	1		1.146	0.7025	0.01	0.1189	7.5149	0.8066	1	0.7164	This study				Novel
NA	NA	NA	NA	NA	NA	NA	NA	NA	NA	NA	NA	NA	NA	NA	NA	NA	NA	NA	NA	NA		NA	NA	NA	NA	NA	NA	NA	NA	This study				Novel
NA	NA	NA	Damaging	0.7508	0.3675	Neutral	0.3043	0.412	31	NA	NA	NA	NA	NA	NA	NA	NA	NA	NA	NA		-0.643	0.04387	0.2669	0.2345	0.845	0.2732	0.7379	0.7379	Henderson RH, Mackay DS, L	2011			
Damaging	-1.66	0.8271	Damaging	0.9692	0.7167	Neutral	0.3203	0.4234	22.7	Benign	0.5019	0.7872	tolerated	0.06	0.5317	Damaging	-3.16	0.6782	probably_damaging	0.952		1.138	0.6469	0.037	0.1602	2.934	0.4865	1	0.7164	This study				Novel
Damaging	-1.79	0.8382	Damaging	0.9564	0.6555	Neutral	0.3626	0.4518	22.9	Pathogeni	0.694	0.8906	deleteriou	0.04	0.4864	Damaging	-4.69	0.8234	probably_damaging	0.999		1.026	0.4595	0.537	0.2786	0.8899	0.2793	0.9549	0.3332	den Hollander AI, Davis J, van	2004	Henderso	2011	
NA	NA	NA	NA	NA	NA	NA	NA	NA	NA	NA	NA	NA	NA	NA	NA	NA	NA	NA	NA	NA		NA	NA	NA	NA	NA	NA	NA	NA	Corton M, Tatu SD, Avila-Fern	2013			
Tolerated	-1.23	0.7886	Damaging	0.9809	0.7959	Damaging	0.7571	0.6962	22.8	Benign	0.6069	0.8475	deleteriou	0.014	0.9125	Damaging	-7.25	0.9505	probably_damaging	1		1.1759	0.7892	0.2879	0.2382	6.26	0.7246	1	0.7164	Khaliq S, Abid A, Hameed A, e	2003	Henderso	2011	
Damaging	-4.98	0.9849	Damaging	0.9972	0.9898	Damaging	0.9146	0.8783	27.4	Pathogeni	0.9649	0.9952	deleteriou	0	0.9125	Damaging	-5.64	0.8691	probably_damaging	1		1.1759	0.7892	0.9959	0.5952	9.336	0.9643	1	0.7164	Henderson RH, Mackay DS, L	2011	Zhang X,	2020	
Damaging	-1.69	0.8299	Damaging	0.9957	0.9755	Damaging	0.8728	0.7948	25.3	Pathogeni	0.8	0.9355	deleteriou	0.003	0.6824	Damaging	-3.61	0.7152	possibly_damaging	0.575		1.138	0.6469	0.9739	0.4595	7.4689	0.7996	0.999	0.4266	Hanein S, Perrault I, Gerber S,	2004			
Damaging	-0.95	0.8608	Damaging	0.9756	0.7558	Damaging	0.6645	0.6336	22.8	Benign	0.3989	0.7163	deleteriou	0.006	0.6144	Damaging	-3.96	0.7506	probably_damaging	0.985		1.312	0.9471	0.842	0.3493	5.6799	0.6783	1	0.7164	Carss KJ, Arno G, Erwood M,	2016			
NA	NA	NA	Damaging	0.9693	0.7169	Neutral	0.3325	0.4319	33	NA	NA	NA	NA	NA	NA	NA	NA	NA	NA	NA		-0.73	0.03703	0.079	0.1857	1.715	0.3759	1	0.7164	This study				Novel
NA	NA	NA	NA	NA	NA	NA	NA	NA	34	NA	NA	NA	NA	NA	NA	NA	NA	NA	NA	NA		NA	NA	NA	NA	NA	NA	NA	NA	Henderson RH, Mackay DS, L	2011			
Damaging	-2.05	0.9136	Damaging	0.9585	0.6644	Damaging	0.5866	0.5846	32	Benign	0.2919	0.6153	tolerated	0.13	0.3516	Damaging	-2.84	0.6158	possibly_damaging	0.562		1.146	0.7025	0.2709	0.2352	1.802	0.3849	1	0.7164	Colombo L, Maltese P E, [illegible]	2021			
Damaging	-3.49	0.9462	Damaging	0.9685	0.7127	Damaging	0.8164	0.7381	23.9	Pathogeni	0.7229	0.9036	Damaging	0.005	0.6323	Damaging	-2.95	0.6436	possibly_damaging	0.837		1.312	0.9471	0.9919	0.541	6.5729	0.7389	1	0.7164	This study				Novel
NA	NA	NA	Damaging	0.8467	0.4376	Neutral	0.3308	0.4307	33	NA	NA	NA	NA	NA	NA	NA	NA	NA	NA	NA		0.09	0.1609	0.9959	0.5952	0.497	0.2222	0.9969	0.4016	Henderson RH, Mackay DS, L	2011			
Damaging	-2.04	0.857	Damaging	0.9818	0.8031	Damaging	0.8685	0.7887	24.5	Pathogeni	0.666	0.8773	deleteriou	0.016	0.6144	Damaging	-2.97	0.6378	benign	0.395		1.1759	0.7892	0.9929	0.5512	7.414	0.7933	1	0.7164	Carss KJ, Arno G, Erwood M,	2016			
NA	NA	NA	Damaging	0.9939	0.9538	NA	NA	NA	32	NA	NA	NA	NA	NA	NA	NA	NA	NA	NA	NA		1.1759	0.7892	0.9679	0.4462	7.0419	0.7629	1	0.7164	Ellingford JM, Barton S, Bhask	2016			
Damaging	-3.96	0.9621	Damaging	0.9971	0.9885	Damaging	0.9389	0.9388	29.8	Pathogeni	0.888	0.9681	deleteriou	0	0.9125	Damaging	-9.29	0.9871	probably_damaging	0.915		1.1759	0.7892	0.972	0.4547	9	0.931	1	0.7164	Tosi J, Tsui I, Lima LH, Wang	2009	Henderso	2011	
Damaging	-1.53	0.8156	Damaging	0.9914	0.9182	Damaging	0.8676	0.7874	25.1	Pathogeni	0.8799	0.9652	deleteriou	0.001	0.7849	Damaging	-3.77	0.7358	probably_damaging	0.998		1.138	0.6469	0.998	0.6591	7.59	0.8192	1	0.7164	Henderson RH, Mackay DS, L	2011			
Tolerated	-0.61	0.7178	Damaging	0.9748	0.7501	Damaging	0.6859	0.6859	25.1	Pathogeni	0.7419	0.9119	deleteriou	0.001	0.7849	Damaging	-5.78	0.8799	possibly_damaging	0.825		1.312	0.9471	0.994	0.563	5.854	0.6924	1	0.7164	Stone EM, Andorf JL, Whitmor	2017			
Tolerated	-1.28	0.7938	Damaging	0.9769	0.7644	Damaging	0.5966	0.5907	26.2	Pathogeni	0.781	0.928	deleteriou	0	0.9125	Damaging	-5.08	0.8289	probably_damaging	0.999		1.138	0.6469	0.924	0.3932	4.762	0.6195	0.9459	0.3289	Henderson RH, Mackay DS, L	2011			
NA	NA	NA	Damaging	0.9919	0.9263	Neutral	0.2952	0.4054	39	NA	NA	NA	NA	NA	NA	NA	NA	NA	NA	NA		1.026	0.4595	0.949	0.4173	7.486	0.8023	1	0.7164	Henderson RH, Mackay DS, L	2011			
Tolerated	-1.31	0.7967	Damaging	0.9803	0.7899	Damaging	0.6627	0.6325	19.01	Benign	0.4269	0.7373	deleteriou	0.03	0.4278	NA	NA	NA	benign	0.132		1.1759	0.7892	0.003	0.08214	5.401	0.6607	1	0.7164	Stone EM, Andorf JL, Whitmor	2017	Henderso	2011	
Tolerated	-0.44	0.6978	Neutral	0.05209	0.1104	Neutral	0.09108	0.1845	21.8	Benign	0.4609	0.7609	deleteriou	0.001	0.7849	Damaging	-5.99	0.9179	possibly_damaging	0.835		0.222	0.2456	0.915	0.3865	0.312	0.1915	0.013	0.1884	Beryozkin A, Zelinger L, Banda	2013			
Tolerated	-0.66	0.7235	Damaging	0.9857	0.8426	Damaging	0.9594	0.9797	25.1	Pathogeni	0.7749	0.9256	deleteriou	0	0.9125	Damaging	-6.84	0.9306	probably_damaging	0.998		1.138	0.6469	0.282	0.2372	7.3949	0.7915	1	0.7164	Motta FL, Salles MV, Costa KA	2017			
NA	NA	NA	NA	NA	NA	NA	NA	NA	NA	NA	NA	NA	NA	NA	NA	NA	NA	NA	NA	NA		NA	NA	NA	NA	NA	NA	NA	NA	This study				Novel
Damaging	-3.95	0.9617	Damaging	0.9888	0.8803	Damaging	0.9573	0.9763	25	Pathogeni	0.9589	0.9936	deleteriou	0	0.9125	Damaging	-10.93	0.9959	probably_damaging	0.994		1.138	0.6469	0.72	0.3139	7.59	0.8192	1	0.7164	Henderson RH, Mackay DS, L	2011			
NA	NA	NA	NA	NA	NA	NA	NA	NA	NA	NA	NA	NA	NA	NA	NA	NA	NA	NA	NA	NA		NA	NA	NA	NA	NA	NA	NA	NA	Henderson RH, Mackay DS, L	2011			
Damaging	-2.9	0.9164	Damaging	0.8717	0.4663	Neutral	0.2082	0.3343	23.4	Benign	0.356	0.6802	deleteriou	0.056	0.4007	NA	NA	NA	probably_damaging	0.965		1.1759	0.7892	0.9549	0.4249	3.3169	0.5168	0.9959	0.3938	This study				Novel
Damaging	-3.42	0.9435	Neutral	0.09596	0.1532	Neutral	0.09597	0.1938	NA	Benign	0.2409	0.5505	Tolerated	0.306	0.2363	NA	NA	NA	NA	NA		0.074	0.1543	0.008	0.1119	-0.0419	0.1202	0	0.06391	Kinga Bujakowska, Isabelle Audo, Saddek Mohand-Saïd [illegible]	2012			
NA	NA	NA	Neutral	0.1173	0.1682	Neutral	0.1366	0.2573	35	NA	NA	NA	NA	NA	NA	NA	NA	NA	NA	NA		0.074	0.1543	0.008	0.1119	-0.0419	0.1202	0	0.06391	This study				Novel
NA	NA	NA	Damaging	0.993	0.9421	Neutral	0.3748	0.4596	39	NA	NA	NA	NA	NA	NA	NA	NA	NA	NA	NA		1.1759	0.7892	0.9929	0.5512	9.493	0.9694	1	0.7164	Li L, Xiao X, Li S, et al. Detect	2011			
NA	NA	NA	NA	NA	NA	NA	NA	NA	16.47	NA	NA	NA	NA	NA	NA	NA	NA	NA	NA	NA		NA	NA	NA	NA	NA	NA	NA	NA	Chan WO, Brennan N, Webste	2020			
Damaging	-6.6	0.9974	Damaging	0.8031	0.3998	Damaging	0.6663	0.6348	25.3	Pathogeni	0.7829	0.9288	deleteriou	0	0.9125	Damaging	-10	0.9882	NA	NA		0.9789	0.3609	0.2689	0.2349	4.98	0.6353	1	0.7164	Siemiatkowska AM, Arimadyo	2011			
NA	NA	NA	NA	NA	NA	NA	NA	NA	33	NA	NA	NA	NA	NA	NA	NA	NA	NA	NA	NA		NA	NA	NA	NA	NA	NA	NA	NA	Hanein S, Perrault I, Gerber S,	2004			
NA	NA	NA	NA	NA	NA	NA	NA	NA	21.8	NA	NA	NA	NA	NA	NA	NA	NA	NA	NA	NA		NA	NA	NA	NA	NA	NA	NA	NA	Cordovez JA, Traboulsi EI, Ca	2015			
NA	NA	NA	Damaging	0.9977	0.9937	NA	NA	NA	34	NA	NA	NA	NA	NA	NA	NA	NA	NA	NA	NA		NA	NA	NA	NA	NA	NA	NA	NA	Cordovez JA, Traboulsi EI, Ca	2015			
NA	NA	NA	Damaging	0.9977	0.9937	NA	NA	NA	34	NA	NA	NA	NA	NA	NA	NA	NA	NA	NA	NA		NA	NA	NA	NA	NA	NA	NA	NA	Coppieters F, Casteels I, Meir	2010			
NA	NA	NA	NA	NA	NA	NA	NA	NA	NA	NA	NA	NA	NA	NA	NA	NA	NA	NA	NA	NA		NA	NA	NA	NA	NA	NA	NA	NA	This study				Novel
Damaging	-2.37	0.8899	Damaging	0.9609	0.6753	Damaging	0.6713	0.6381	24.8	Pathogeni	0.7289	0.9062	deleteriou	0	0.9125	Damaging	-3.53	0.7007	probably_damaging	0.988		1.0169	0.3893	0.5939	0.2883	3.173	0.5054	1	0.7164	This study				Novel
Damaging	-2.33	0.8791	Damaging	0.8791	0.4326	Neutral	0.2984	0.4077	19.56	Benign	0.5569	0.8199	deleteriou	0	0.9125	NA	NA	NA	possibly_damaging	0.748		0.9179	0.3324	0.012	0.1245	1.585	0.3621	0.9739	0.3463	Henderson RH, Mackay DS, L	2011			
Damaging	-2.11	0.8845	Damaging	0.9965	0.9832	Damaging	0.9965	0.9832	27	Pathogeni	0.665	0.8768	deleteriou	0	0.9125	Damaging	-2.55	0.9081	probably_damaging	1		0.976	0.976	0.87	0.3609	7.697	0.8351	1	0.7164	This study				Novel
Damaging	-2.37	0.8885	Damaging	0.9961	0.9793	Damaging	0.8903	0.8251	28.5	Pathogeni	0.6999	0.8933	deleteriou	0	0.9125	Damaging	-3.08	0.9294	probably_damaging	1		0.976	0.976	0.87	0.3609	7.697	0.8351	1	0.7164	Henderson RH, Mackay DS, L	2011			
NA	NA	NA	NA	NA	NA	NA	NA	NA	NA	NA	NA	NA	NA	NA	NA	NA	NA	NA	NA	NA		NA	NA	NA	NA	NA	NA	NA	NA	This study				Novel
NA	NA	NA	NA	NA	NA	NA	NA	NA	NA	NA	NA	NA	NA	NA	NA	NA	NA	NA	NA	NA		NA	NA	NA	NA	NA	NA	NA	NA	This study				Novel
