# Supplementary material for: CRB1-Associated Retinal Dystrophies: Genetics, Clinical Characteristics, and Natural History
Source: Am J Ophthalmol. 2023 Feb;246:107–21. doi: 10.1016/j.ajo.2022.09.002 (PMC10555856; doi:10.1016/j.ajo.2022.09.002)
Supplement: Supplementary file 5 [file mmc5.pdf]

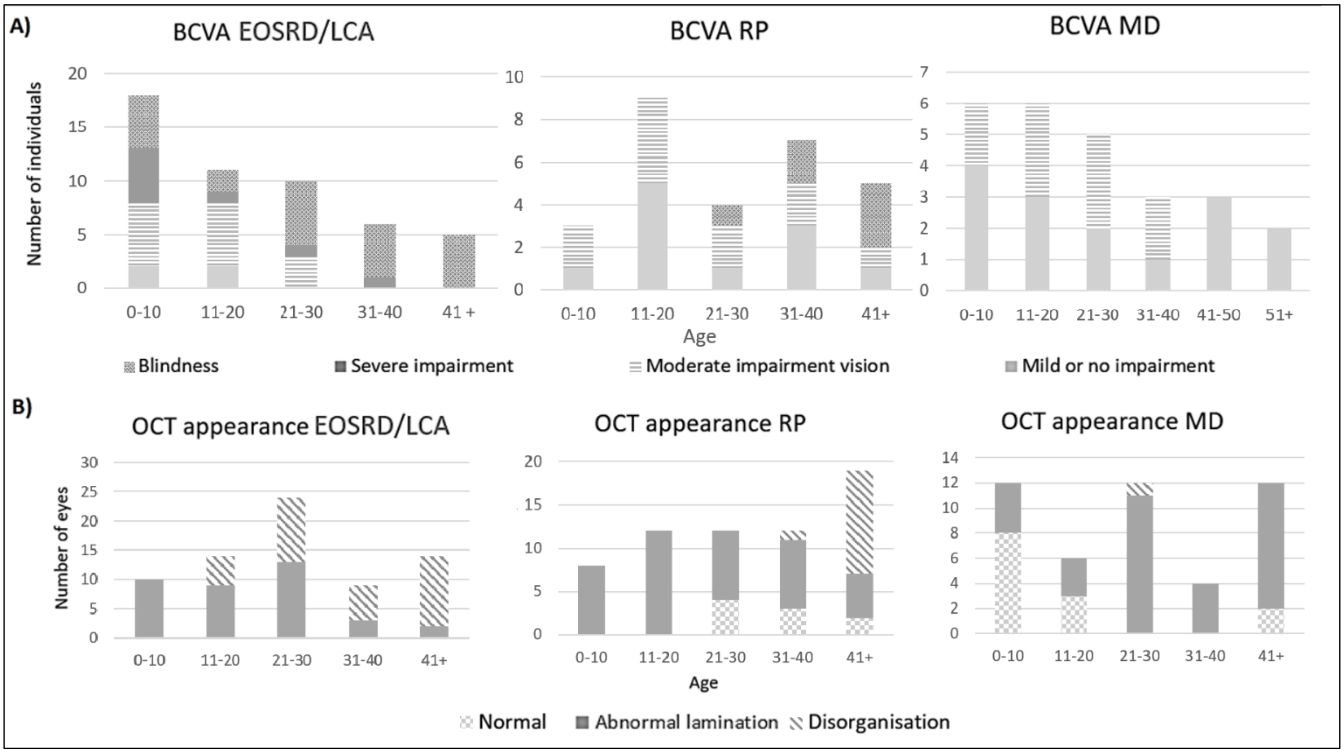

Supplementary figure 1. Classification of individuals from the different subgroups according to their latest best corrected visual acuity (BCVA) and the macular OCT group. A) Each subgroup is divided both into age groups and into the WHO categories of normal vision to mild vision impairment, moderate and severe visual loss, and blindness. B) Macular OCT is divided into three categories of normal, ill-defined lamination, and disorganisation. Individuals from each sub-cohort are grouped by age and subsequently according to their OCT appearance.
