# Supplementary material for: CRB1-Associated Retinal Dystrophies: Genetics, Clinical Characteristics, and Natural History
Source: Am J Ophthalmol. 2023 Feb;246:107–21. doi: 10.1016/j.ajo.2022.09.002 (PMC10555856; doi:10.1016/j.ajo.2022.09.002)
Supplement: Supplementary file 6 [file mmc6.pdf]

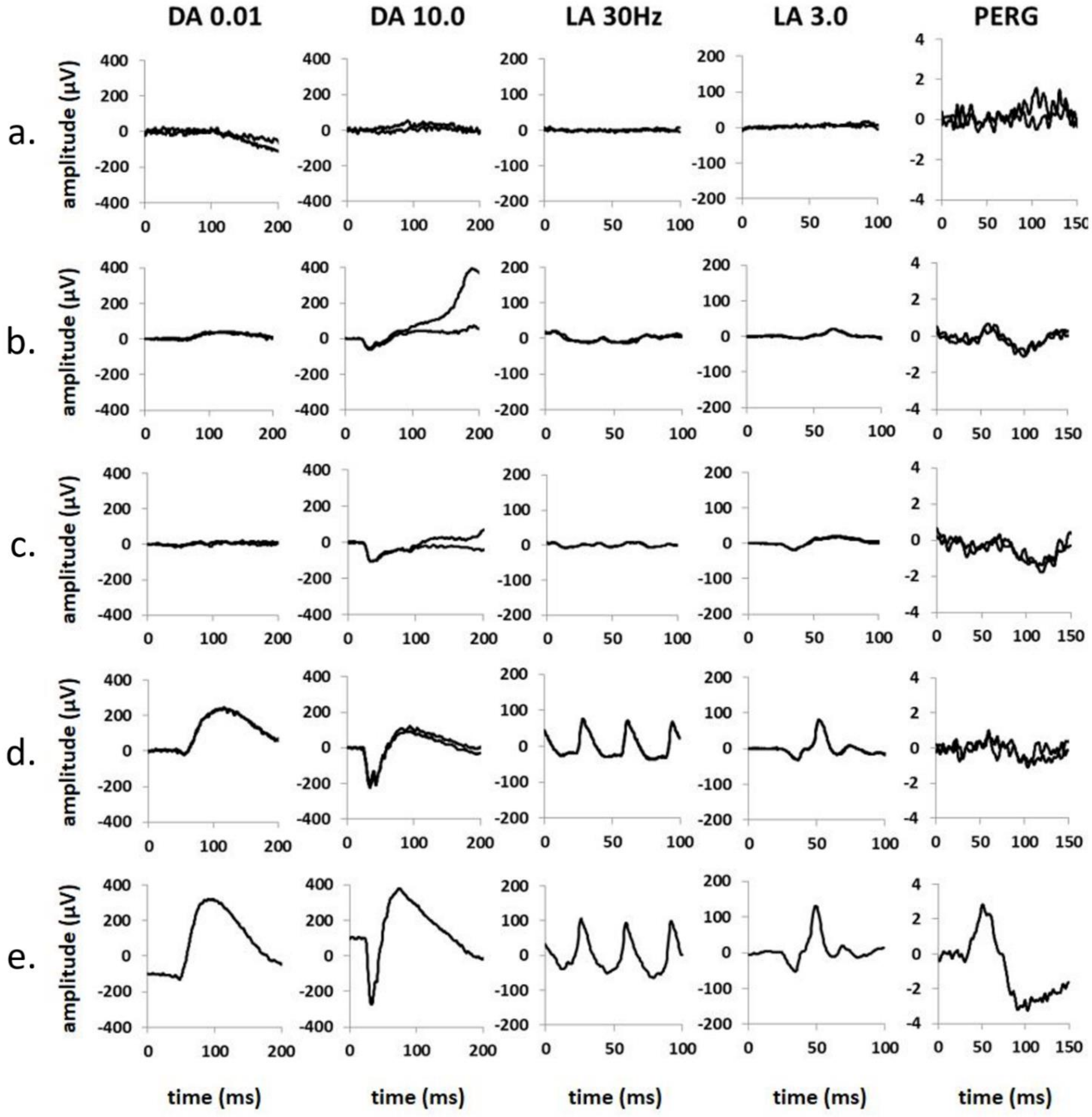

Supplementary figure 2. Representative full-field and pattern ERGs from patients 9 (a; LCA, aged 15 years), 12 (b; RP, 11 years), 23 (c; RP, aged 21 years) and 16 (d; MD, 43 years), corresponding to the patient numbering used in Figure 5. Representative control ("normal") recordings are shown for comparison (e). Data are shown for the right eyes only, as all showed a high degree of inter-ocular symmetry. Patient traces are

superimposed to demonstrate reproducibility. a) ERGs are undetectable consistent with a severe loss of rod and cone photoreceptor function and a clinical diagnosis of LCA; b) DA and LA ERGs show a similar degree of attenuation consistent with a photoreceptor dystrophy; c) the LA ERGs are proportionately slightly worse than the DA ERGs, suggestive of a cone-rod dystrophy; d) the ERGs reveal no definite abnormality but pattern ERG P50 is subnormal, in keeping with macular dystrophy.
