# Supplementary material for: CRB1-Associated Retinal Dystrophies: Genetics, Clinical Characteristics, and Natural History
Source: Am J Ophthalmol. 2023 Feb;246:107–21. doi: 10.1016/j.ajo.2022.09.002 (PMC10555856; doi:10.1016/j.ajo.2022.09.002)
Supplement: Supplementary file 7 [file mmc7.pdf]

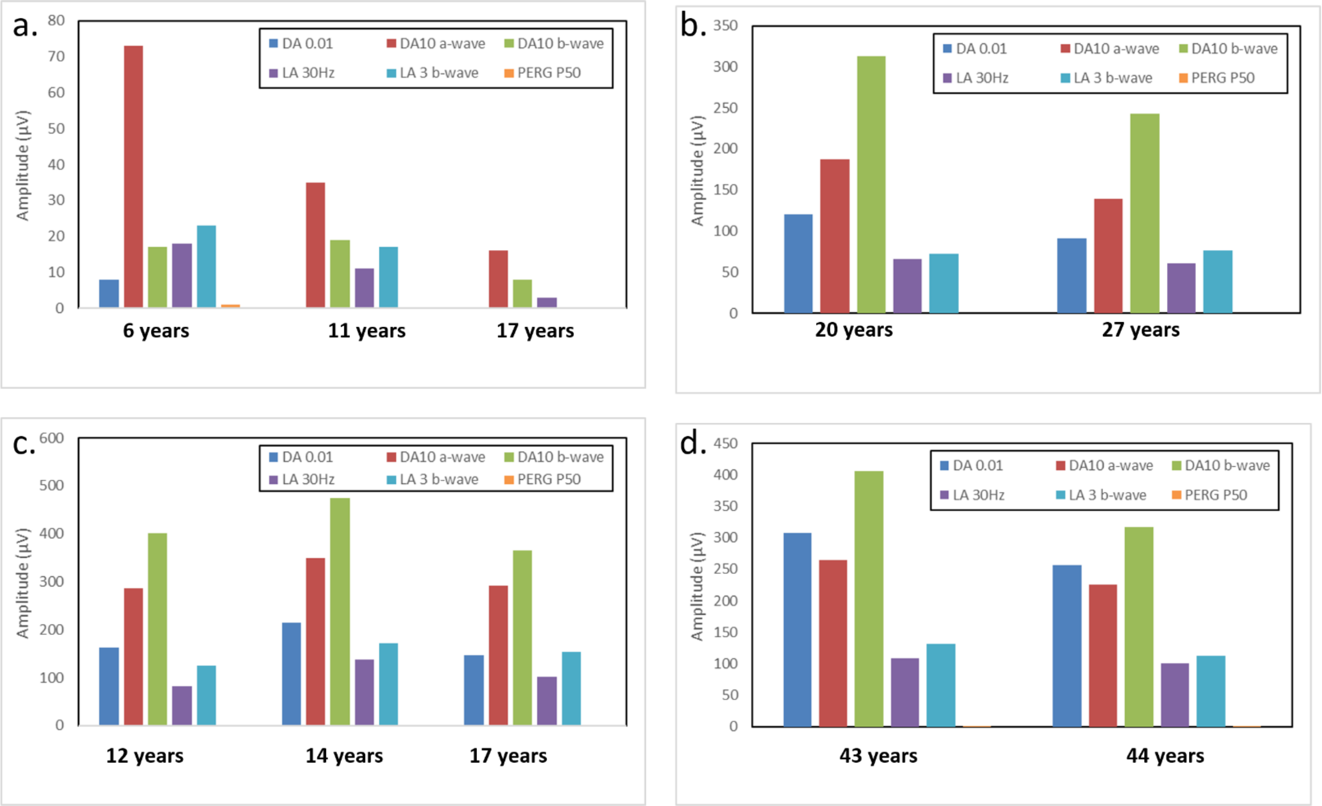

Supplementary figure 3. Comparison of the main ERG amplitude parameters at baseline with those obtained at follow-up for patients 14 (a, LCA), 22 (b, MD), 25 (c, MD) and 23 (d, MD), corresponding to the patient numbering in Figure 5 (main manuscript) Ages at the time of testing are shown on the x-axis. Pattern ERGs were subnormal at baseline in patient 14 (a), and were otherwise undetectable on all occasions in all 4 subjects. Note the stability of ERGs in b) and d), consistent with a macular dystrophy.
