# Supplementary material for: CRB1-Associated Retinal Dystrophies: Genetics, Clinical Characteristics, and Natural History
Source: Am J Ophthalmol. 2023 Feb;246:107–21. doi: 10.1016/j.ajo.2022.09.002 (PMC10555856; doi:10.1016/j.ajo.2022.09.002)
Supplement: Supplementary file 8 [file mmc8.pdf]

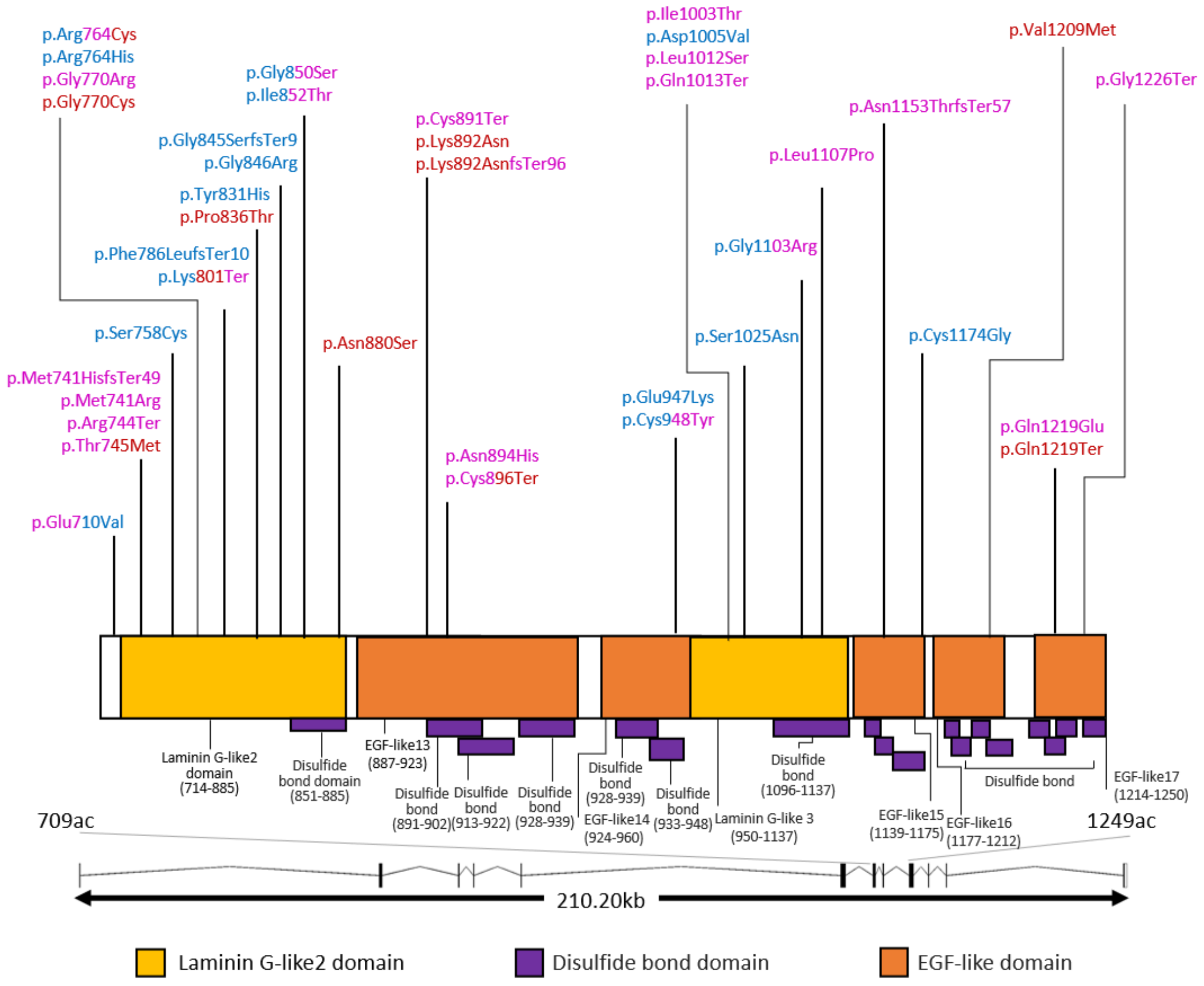

Supplementary figure 4. Graphic representation of exons 7-9 of *CRB1* gene and protein, with details on functional domains. Each variant within our cohort is detailed above the protein, with its corresponding location, and with different colors according to the phenotype they were found associated with.
