# Supplementary material for: CRB1-Associated Retinal Dystrophies: Genetics, Clinical Characteristics, and Natural History
Source: Am J Ophthalmol. 2023 Feb;246:107–21. doi: 10.1016/j.ajo.2022.09.002 (PMC10555856; doi:10.1016/j.ajo.2022.09.002)
Supplement: Supplementary file 9 [file mmc9.pdf]

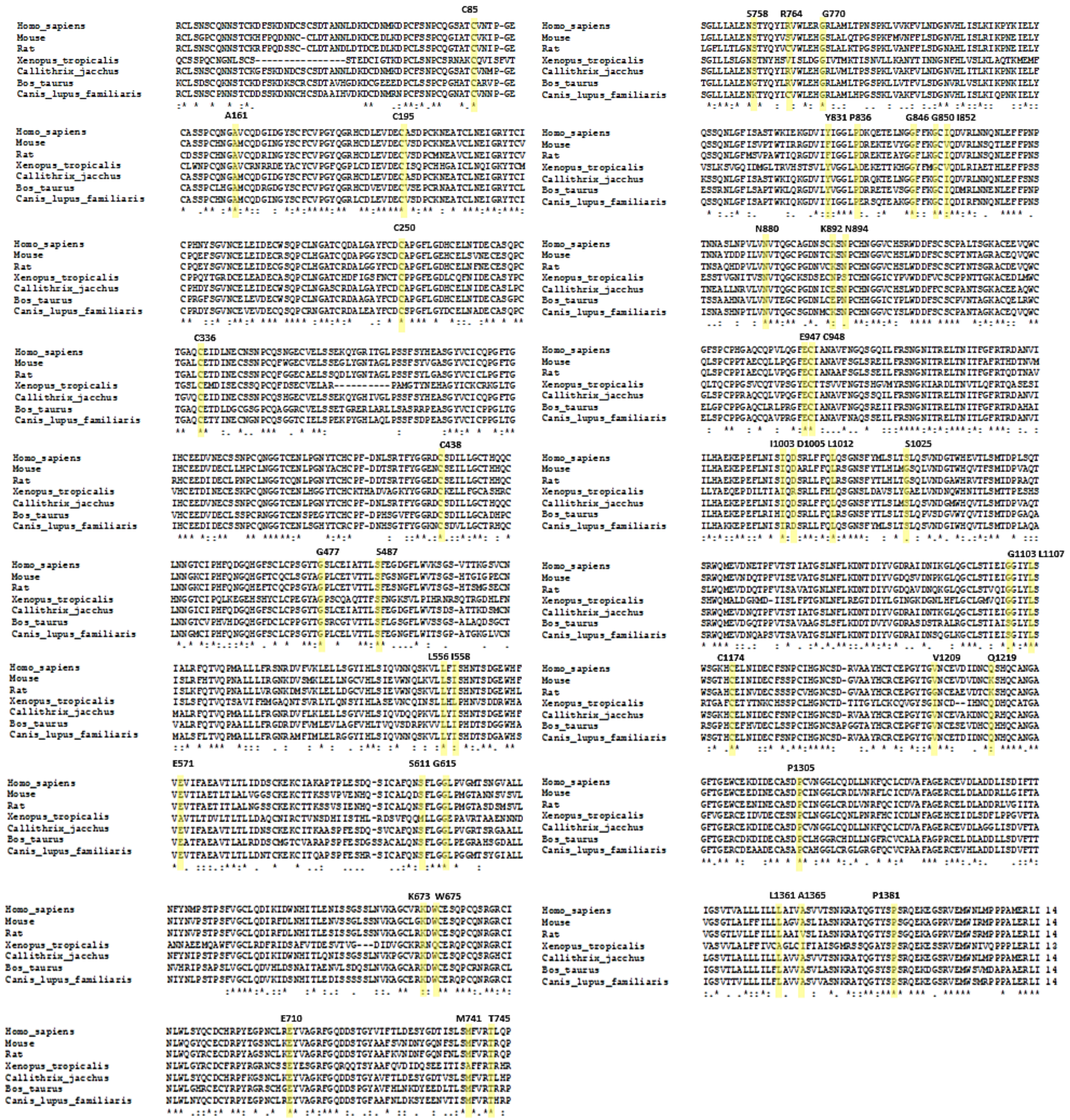

Supplementary figure 5. Multiple alignment of 7 species of CRB1. Evolutionary conservation of the affected amino acid residues was evaluated with Clustal Omega software (http://www.ebi.ac.uk/Tools/msa/clustalo/; accessed on 1st November 2021) and the amino acid sequence alignment was numbered in accordance with the Homo sapiens CRB1 sequence (ENST00000367400.3). An asterisk indicates complete conservation across the 7 species.
